# Revision of the calcareous fen arachnofauna: habitat affinities of the fen-inhabiting spiders

**DOI:** 10.3897/zookeys.802.26449

**Published:** 2018-12-04

**Authors:** Maija Štokmane, Inese Cera

**Affiliations:** 1 University of Latvia, Faculty of Biology, Department of Zoology and Animal Ecology, Jelgavas street 1, LV–1004, Riga, Latvia University of Latvia, Faculty of Biology Riga Latvia; 2 University of Latvia, Institute of Biology, Laboratory of Bioindication, Miera street 3, LV–2169, Salaspils, Latvia University of Latvia, Institute of Biology Salaspils Latvia

**Keywords:** Araneae, ecological groups, Latvia, mire habitats, spider fauna

## Abstract

Calcareous fens are one of the most species-rich habitats of the temperate zone of the Northern Hemisphere. In spite of this species richness, however, calcareous fens are still rather poorly investigated. Consequently, the data of the fen-associated spider fauna are also largely lacking. The aim of the research was to study the spider fauna of the calcareous fens of Latvia and to draw conclusions about what kind of spider species and ecological groups typically inhabit calcareous fen habitats. Spiders were sampled in the summer months of 2010, 2011, and 2012 at nine different calcareous fens of the coastal lowland of Latvia. The spider collection was performed by pitfall traps and a sweep net. The examined material comprised 6631 adult spider individuals representing 21 families and 149 species. The main spider ecological groups that dominated in the studied calcareous fens were hygrophilous and photophilous species which largely reflect the main properties of our studied habitats, all of which were wet, open mire habitats. Nevertheless, the fen arachnofauna consisted also of spider groups which are less typical for moist, sun-exposed, and alkaline environments, like xerophilous, sciophilous, and sphagnophilous species, respectively. Finally, several spider species collected in this study have not been previously reported for the spider fauna of Latvia, and many more might still be undiscovered in these unique and poorly investigated habitats. Therefore, it is suggested that calcareous fens deserve special attention and they should definitely be investigated further.

## Introduction

Mire habitats (fens and bogs) are among the most important wetland ecosystems of Europe. They are characterised by specialized flora and fauna and the presence of specially protected species (Bambe et al. 2008; Auniņš et al. 2013). In contrast to bogs, fen habitats are rather poorly investigated, the same being applied to their arachnofauna. There are only very few studies in Europe regarding the spider fauna of fens – we could find only a single study from Latvia (Cera et al. 2010), as well as one study from Estonia (Vilbaste 1980) and one from Poland (Kajak et al. 2000). Bogs are much more popular habitats for arachnological studies – there are several studies from Latvia (Šternbergs 1991; Spuņģis 2008), as well as from Estonia (Vilbaste 1980), Lithuania (Rėlys and Dapkus 2002b; Rėlys et al. 2002; Biteniekyté and Rélys 2006, 2008), Poland (Kupryjanowicz et al. 1998), Finland (Koponen 2002a,b, 2003, 2004), Norway (Pommeresche 2002), Denmark (Bruun and Toft 2004), Germany (Buchholz 2016), Russia (Oliger 2004), Romania (Urák and Samu 2008; Samu and Urák 2014) and other countries (Štambuk and Erben 2002; Scott et al. 2006).

Although both fens and bogs are mire habitats, there are several fundamental differences between them: (1) fens are mires that receive water and nutrients from groundwater and/or surface water, as well as from rainfall, while bogs depend solely on precipitation (McBride et al. 2011); (2) fens are mineral-rich type of mires which are usually characterized by basic or circumneutral conditions, while bogs are nutrient poor mires which have strongly acidic (pH < 5.0) soil conditions (Kellner 2003; Spitzer and Danks 2006; Horsák et al. 2011); (3) fens are dominated by brown mosses and sedges (e.g., *Carex*, *Cladium*, *Schoenus*), while bogs – by peat mosses (*Sphagnum* spp.) (Rydin and Jeglum 2006; Gałka et al. 2016); and (4) fens are rich in a floristic sense, while bogs have a low species diversity (Kellner 2003; McBride et al. 2011). Because of these differences between fens and bogs, and because of the fact that fens are much less studied than bogs, it is clear that more studies are needed in fen habitats.

Calcareous fens are one of those fen types which are especially worth studying because they belong to the most species-rich ecosystems of the temperate zone of the Northern Hemisphere (Joosten and Clarke 2002). In addition, there are some plant and animal species that occur almost exclusively in this habitat type. For example, *Scorpidiumcossonii*, *Schoenusferrugineus*, *Carexdavalliana*, *Ophrysinsectifera*, *Saussureaesthonica* and *Juncussubnodulosus* are plant species that can be found only within calcareous fens (Auniņš et al. 2013). Also, calcareous fens is a very important habitat for specially protected snail species, such as *Vertigogenesii* and *V.geyeri* (Cameron et al. 2003; Auniņš et al. 2013). Overall, calcareous fens are very rare in most of the countries in the European Union (Stanová et al. 2008), and they are considered priority habitats in Annex I of the EU Habitat Directive (EC 1992). Thus, because of the rarity of the calcareous fens and because of the presence of unique species within these habitats, it would be important and worthwhile to assess the quality of the calcareous fens, as well as to investigate their flora and fauna.

Spiders have been shown to be very good bioindicators (e.g., Marc et al. 1999; Pearce and Venier 2006), and thereby they are proposed as a group of organisms that are potentially useful tools for assessing the conservation value of rare and threatened habitats. Unfortunately, arachnids from calcareous fens are very poorly studied. In Latvia, spiders within calcareous fens have been investigated in some of our previous studies (Štokmane et al. 2013; Štokmane and Spuņģis 2014, 2016). These previous investigations had examined the influence of vegetation structure on spider diversity, while little attention was paid on the faunistic aspects of the fen spider communities. Thereby, the main purpose of the present study was to investigate the spider fauna of calcareous fens in greater detail and to analyse what kind of spider species and ecological groups are more typical for this habitat type.

## Materials and methods

**Figure 1.**
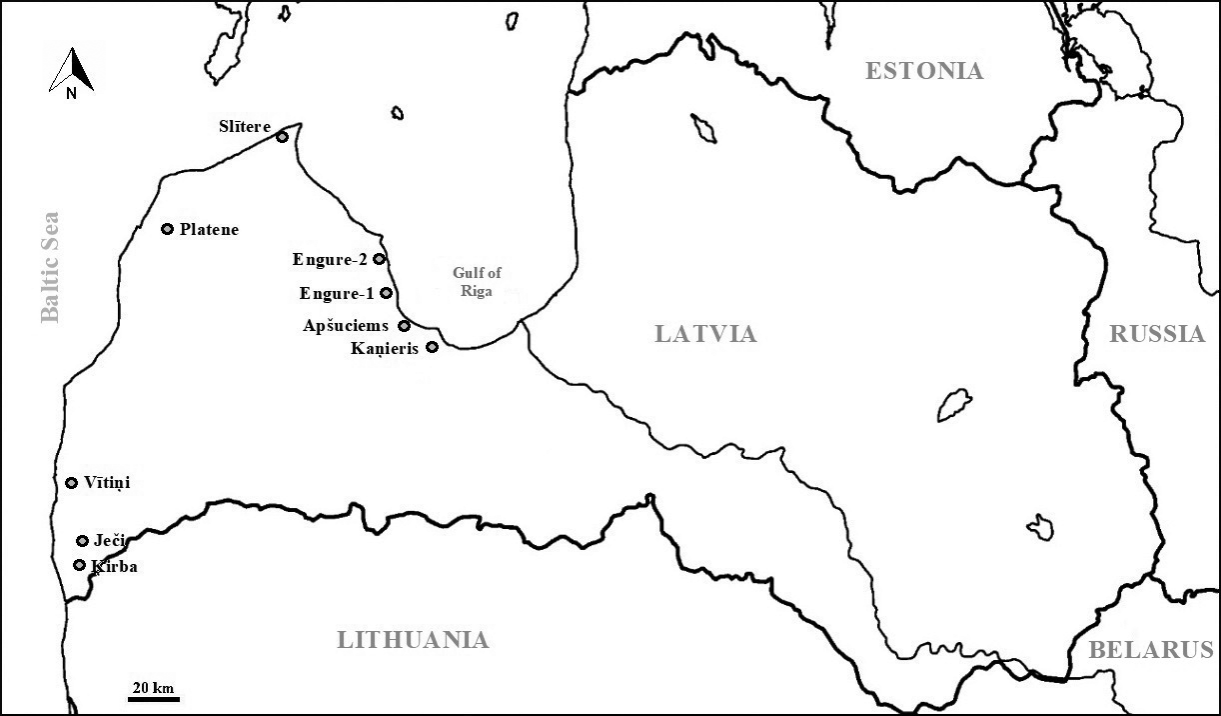
Map showing the calcareous fens studied (marked with circles). All fens are located in the coastal lowland of Latvia.

The present study is a compilation and an overview of our three previous studies made in the summers of the following years: 2010, 2011, and 2012. These studies were carried out in nine different calcareous fens of the coastal lowland of Latvia: (1) Kaņieris; (2) Apšuciems; (3) Engure-1; (4) Engure-2; (5) Slītere; (6) Platene; (7) Vītiņi; (8) Ječi; and (9) Ķirba (Figure 1). All the studied fens belong to the EU *Natura 2000* network.

A short overview of the used sampling methods is given in Table 1. As it can be seen, the spider capture methods as well as the time of sampling differed in each of the three study years. This was done in order to access more spider species and enlarge the species list, since it is well known that, firstly, each collecting method targets different spider species (e.g., Churchill and Arthur 1999) and, secondly, spiders exhibit seasonal variation in their occurrence (e.g., Marc et al. 1999). The sampling period of the present study, however, was limited to the summer months only (June/July/August). We decided that summer will be the optimal time for collecting spiders since it is the warmest season in Latvia (LEGMC 2014) when vegetation biomass is at its prime and food resources are plentiful, and thus we assumed that spider diversity as well as the number of species and individuals will be much greater at this period of time. Higher abundance and diversity of spiders during summer is also consistent with the findings of other researchers (e.g., Hatley and Macmahon 1980; Reddy and Venkataiah 1986).

**Table 1.** A short overview of the used methods in each of the three study years. There were two different calcareous fens chosen at the wetlands of the lake Engure in 2010 (designated as Engure-1 and Engure-2).

Data	2010	2011	2012
**Number of studied fens**	5	8	1
**The studied fens**		Kaņieris	Apšuciems
		Apšuciems	
	Kaņieris	Engure	
	Apšuciems	Slītere	
	Engure-1	Platene	
	Platene	Vītiņi	
		Ječi	
		Ķirba	
**Spider sampling methods**	Pitfall trapping	Sweep netting	Pitfall trapping & sweep netting
**Sampling dates**	5 June – 3 July (in Platene: 6 June – 4 July)	16 – 17 July	Traps: 27 July – 22 August; Sweeping: 26 – 27 July
**Detailed information on the methods**	Štokmane et al. (2013)	Štokmane and Spuņģis (2014)	Štokmane and Spuņģis (2016)

A binocular microscope at 45× magnification was used to identify the spiders to species level. The unidentified adult species were recorded as morphospecies. Since juveniles could be identified only to family level, they were excluded from the study. The full species list of calcareous fen spiders as well as the number of collected individuals in each fen can be found in Appendix 1. The nomenclature of spider species followed the World Spider Catalog (2018).

In order to understand what kind of spider species inhabit calcareous fens, we carried out a literature survey and prepared a short description on the habitat preferences for each of our collected spider species. The habitat affinities of the spider species were derived from many different literature sources, but mainly from Locket and Millidge (1951, 1953), Roberts (1996), Harvey et al. (2002a,b), Almquist (2005, 2006), Matveinen-Huju et al. (2006), Oxbrough et al. (2006), Nentwig et al. (2012)and Arachnologische Gesellschaft (2018). Based on the literature analysis, all the collected spider species were sorted into a number of ecological groups. These groups were distinguished mainly by taking into account the spider requirements for moisture and light, since these two abiotic factors are among the most important determinants characterizing the habitats of spiders (Entling et al. 2007). When taking into account the moisture preferences, the species were classified as either being hygrophilous (water-loving) or xerophilous (drought-loving), but when taking into account the light preferences, the species were classified as either being photophilous (sun-loving) or sciophilous (shade-loving). Species with a wide ecological amplitude (= found in many different habitat types) were classified as being habitat generalists.

In addition, we have summarized the information whether our detected spider species have been found within other European mires. We have chosen to include in our summary those mire studies in which the full spider species list has been published. Thus, we included the following studies: Cera et al. (2010) (calcareous fens of Latvia), Šternbergs (1991) (Baži bog of Latvia), Kajak et al. (2000) (fens of Poland), Kupryjanowicz et al. (1998) (bogs of Poland), Vilbaste (1980) (fens and bogs of Estonia), Koponen (2002a,b) (bogs of Northern Europe, including Sweden, Finland and northern Norway), Rėlys and Dapkus (2002b) (bogs of Lithuania), and Rėlys et al. (2002) (bogs of Lithuania and Finland). This information is presented as presence data in Appendix 2.

All our collected spider specimens are stored in 70% ethanol, labelled, and deposited in the Department of Zoology and Animal Ecology, Faculty of Biology, University of Latvia, Riga.

## Results

Overall, in the three study years a total number of 8,967 spider individuals (6631 adults and 2336 juveniles) were collected, representing 134 species and 15 morphospecies in 21 families. Most of the species (87 spp.) were collected only in a single year, while only five species were detected in all three study years (*Dolomedesfimbriatus*, *Evarchaarcuata*, *Tibellusmaritimus*, *Xysticusulmi* and *Kaestneriapullata*). Altogether eight spider species found during this investigation were registered as new species for the araneofauna of Latvia – *Cheiracanthiumpunctorium* (Eutichuridae), *Gnaphosalapponum* (Gnaphosidae), *G.nigerrima* (Gnaphosidae), *Bathyphantesparvulus* (Linyphiidae), *Centromerussemiater* (Linyphiidae), *Microlinyphiaimpigra* (Linyphiidae), *Piratatenuitarsis* (Lycosidae), and *Leptorchestesberolinensis* (Salticidae). The dominant spider species in each year and in each fen are given in Table 2. One of the most abundant and most frequently recorded species was *Dolomedesfimbriatus*, which occurred in the vast majority of the studied calcareous fens. Overall, however, there were rather large differences in spider species composition between fens, between study years, as well as between pitfall samples and the sweep-net samples.

**Table 2.** The most abundant (>9.5%) spider species in each of the studied fens in each study year. Two of the fens (Vītiņi and Slītere) are not given here because too few spiders were collected within these fens.

	Apšuciems		Engure-1	Engure-2	Ječi	Kaņieris	Ķirba	Platene
**Pitfall trapping**	**2010**	**2012**	**2010**	**2010**		**2010**		**2010**
* Antistea elegans *	30.2	10.1		16.2				
* Bathyphantes parvulus *	9.5							
*Centromerus* sp.						11.6		
* Hygrolycosa rubrofasciata *		9.5						
* Pardosa prativaga *								36.4
* Pardosa pullata *				13.2				
* Pirata tenuitarsis *								13
* Piratula uliginosa *				15		34.7		
* Piratula hygrophilus *		9.9						
* Piratula knorri *								12.5
* Piratula latitans *			56.2					
* Trochosa terricola *		11.8						
* Zora spinimana *		9.6						
**Sweep netting**	**2011**	**2012**	**Engure 2011**		**2011**	**2011**	**2011**	**2011**
* Dolomedes fimbriatus *	19	59.5	33.3		19.5	39.1	41.2	37.5
* Evarcha arcuata *	26.2	13.1				10.1		
* Tibellus maritimus *	21.4		57.1		58.5	17.4	32.4	37.5

By using relevant information from the literature (see the method section), we have prepared a short description of each of the collected spider species (Table 3). Also, all the collected spider species were sorted into ecological groups according to their habitat requirements. This classification was based mainly on spider requirements for moisture (hygrophilous/xerophilous species) and light (photophilous/sciophilous species). For some of the spider species we distinguished also sub-groups. In some cases, however, it was difficult to classify a spider species into a particular ecological group(-s), because the habitat preferences of some spiders are rather poorly defined (Eyre and Woodward 1996), and the information in the literature is sometimes contradictory (personal observation).

**Table 3.** List of spider species collected in the calcareous fens of Latvia and description of their habitat preferences. The ecological group(s) of each species are also indicated (**bold**). For some of the species the ecological sub-group is given as well. Genera and species are sorted alphabetically within each family.

Family	Species	Description of the species habitat preferences	Ecological group(s)
Agelenidae	*Agelenalabyrinthica* (Clerck, 1757)	It can be found in habitats such as sandy heathlands, banks of ditches (Almquist 2005), sunny forest edges (Nentwig et al. 2012), grasslands (Harvey et al. 2002b), bogs (Vilbaste 1980). This species can also occur in coastal sites – it has been found in coastal dune habitats in Latvia (Cera and Spuņģis 2013), as well as in salt marshes at the North Sea coast (Finch et al. 2007).	Hygrophilous | Xerophilous; **Photophilous** | Sciophilous; Habitat generalist
Araneidae	*Araneusalsine* (Walckenaer, 1802)	It is usually found in damp, sheltered woodland clearings (Roberts 1996; Harvey et al. 2002b). It can also be found in humid forest edges, damp meadows, bogs (Vilbaste 1980; Almquist 2005; Nentwig et al. 2012).	**Hygrophilous** | Xerophilous; Photophilous | Sciophilous; Habitat generalist
	*Araneusdiadematus* Clerck, 1757	It is one of the most common and abundant species (Locket and Millidge 1953) which is rather ubiquitous (Cattin et al. 2003) – it occurs wherever the habitat can provide supports for its large orb web (Harvey et al. 2002b). It can be found in a wide range of habitats, such as all types of woodland, grasslands, hedgerows, heathland, as well as roadside verges, quarries, gardens, buildings and different other places (Nyffeler and Benz 1987; Harvey et al. 2002b; Almquist 2005; Arachnologische Gesellschaft 2018). It, however, seems to prefer forest edges and gardens (Heimer and Nentwig 1991; Nentwig et al. 2012).	Hygrophilous | Xerophilous; **Photophilous** | Sciophilous; Habitat generalist *Sub-group*: Ecotonal forest species
	*Araneusquadratus* Clerck, 1757	It occurs in grasslands (Nyffeler and Benz 1987, 1989; Harvey et al. 2002b), especially in moist meadows (Almquist 2005; Nentwig et al. 2012). This species is found on vegetation which has sufficient height and strength to support its large orb web, such as tall grasses, heather and bushes such as gorse (Roberts 1996; Harvey et al. 2002b). *A.quadratus* can also be found in bogs (Vilbaste 1980; Almquist 2005) and fens (Vilbaste 1980; Kajak et al. 2000; Cera et al. 2010).	Hygrophilous | Xerophilous; **Photophilous** | Sciophilous; Habitat generalist *Sub-group*: Grassland species
	*Araniellacucurbitina* (Clerck, 1757)	It is found in a wide variety of situations, for example, in broadleaved deciduous woodland, dry grasslands, hedgerows, thermophile woodland fringes etc. (Arachnologische Gesellschaft 2018). Most commonly, however, the species is found on trees and bushes in woodland, scrub and hedgerows, as well as on nearby low vegetation (Roberts 1996; Harvey et al. 2002b; Nentwig et al. 2012). Harvey et al. (2002b) wrote that the main tree that is inhabited by *A.cucurbitina* is oak, however Almquist (2005) mentions also pine, spruce and birch.	Hygrophilous | Xerophilous; Photophilous | Sciophilous; **Habitat generalist**
	*Argiopebruennichi* (Scopoli, 1772)	It is obviously associated with different grassland habitats (Nyffeler and Benz 1987, 1989; Harvey et al. 2002b; Spungis 2005; Horváth et al. 2009), especially with moist meadows (Almquist 2005; Nentwig et al. 2012). This species has also been found in gardens, wasteland, wetlands, roadside verges and on house walls (Harvey et al. 2002b; Cattin et al. 2003; Almquist 2005).	Hygrophilous | Xerophilous; **Photophilous** | Sciophilous; Habitat generalist *Sub-group*: Grassland species
	*Larinioidescornutus* (Clerck, 1757)	This species usually inhabits damp places (Harvey et al. 2002b; Cattin et al. 2003). It occurs in wetlands (Kajak et al. 2000; Cattin et al. 2003; Cera et al. 2010), as well as in meadows and forest edges, mostly near water (Harvey et al. 2002b; Almquist 2005; Nentwig et al. 2012).	**Hygrophilous** | Xerophilous; Photophilous | Sciophilous; Habitat generalist
	*Mangoraacalypha* (Walckenaer, 1802)	It occurs in open woodland, heathland, dry meadows, dune areas and in many other places (Harvey et al. 2002b; Almquist 2005; Arachnologische Gesellschaft 2018). Rėlys and Dapkus (2002) found *M.acalypha* in a pine bog, but Kajak et al. (2000) – in fens. Overall, however, the species seems to prefer warm, dry and sunny places (Harvey et al. 2002b; Nentwig et al. 2012).	Hygrophilous | **Xerophilous**; **Photophilous** | Sciophilous; Habitat generalist
Araneidae	*Neosconaadianta* (Walckenaer, 1802)	It is associated with dry and warm places and can be found in a range of open habitats (Harvey et al. 2002b; Arachnologische Gesellschaft 2018). The species occurs, for example, in heathlands (Harvey et al. 2002b; Almquist 2005), grasslands (Harvey et al. 2002b; Horváth et al. 2009; Cera 2013; Arachnologische Gesellschaft 2018), screes and in other sparsely vegetated habitats (Arachnologische Gesellschaft 2018). *N.adianta* can, however, also be found in marshy areas – in fens and saltmarshes (Kajak et al. 2000; Harvey et al. 2002b; Nentwig et al. 2012).	Hygrophilous | **Xerophilous**; **Photophilous** | Sciophilous; Habitat generalist
	*Singahamata* (Clerck, 1757)	It occurs in damp habitats (Roberts 1996; Harvey et al. 2002b), e.g., moist meadows and pastures, reed-beds, fens, bogs, etc. (Vilbaste 1980; Kajak et al. 2000; Harvey et al. 2002b; Almquist 2005; Arachnologische Gesellschaft 2018). The species prefers sunny places – along with the already mentioned open habitats, it can also be found, for example, in open woods, ruderal areas and waysides (Almquist 2005; Nentwig et al. 2012).	**Hygrophilous** | Xerophilous; **Photophilous** | Sciophilous; Habitat generalist
Clubionidae	*Clubionagermanica* Thorell, 1871	It can be found on trees and shrubbery of different habitats, especially in forests and parks (Miller 1971; Nentwig et al. 2012). Komposch (2000) has found this species in alder forest, while Almquist (2006) proposes that this species can be found in damp deciduous woods and damp shores with bog-myrtle *Myricagale*. This spider species can also be found in hedgerows, reed-beds (Arachnologische Gesellschaft 2018), fens and bogs (Vilbaste 1980).	**Hygrophilous** | Xerophilous; Photophilous | Sciophilous; Habitat generalist
	*Clubionareclusa* O. Pickard-Cambridge, 1863	It occurs in a wide range of habitats (Roberts 1996; Harvey et al. 2002b), however most often it can be found in damp or marshy places (Harvey et al. 2002b). It occurs in marshes, borders of bogs, damp meadows, on water borders etc. (Vilbaste 1980; Almquist 2006; Nentwig et al. 2012).	**Hygrophilous** | Xerophilous; Photophilous | Sciophilous; Habitat generalist *Sub-group*: Hygrophilous generalist
	*Clubionastagnatilis* Kulczyński, 1897	It occurs in different damp and marshy situations – in swamps, fens, bogs, shores of lakes, reed-beds etc. (Locket and Millidge 1951; Vilbaste 1980; Kajak et al. 2000; Harvey et al. 2002b; Rėlys and Dapkus 2002; Almquist 2006; Arachnologische Gesellschaft 2018). The species might also be found in damp woodlands (Nentwig et al. 2012).	**Hygrophilous** | Xerophilous; Photophilous | Sciophilous; Habitat generalist *Sub-group*: Hygrophilous generalist
	*Clubionasubsultans* Thorell, 1875	It is associated mostly with pine or spruce forests (Roberts 1996; Harvey et al. 2002b; Duffey 2005; Matveinen-Huju et al. 2006; Nentwig et al. 2012; Arachnologische Gesellschaft 2018). This species is found on branches, as well as on and under bark of conifers and in pine litter amongst pine needles (Roberts 1996; Harvey et al. 2002b). Vilbaste (1980) has found this species in fens and bogs of Estonia.	Hygrophilous | Xerophilous; Photophilous | **Sciophilous**; Habitat generalist *Sub-group*: Coniferous forest species
Dictynidae	*Argennasubnigra* (O. Pickard-Cambridge, 1861)	It occurs in sunny, sparsely vegetated localities (Harvey et al. 2002b; Bonte et al. 2004; Nentwig et al. 2012). It is mainly found in dry grasslands, coastal dunes, old quarries, wasteground and railings (Locket and Millidge 1951; Harvey et al. 2002b; Duffey 2005; Almquist 2006; Arachnologische Gesellschaft 2018). Was found in fens by Kajak et al. (2000).	Hygrophilous | **Xerophilous**; **Photophilous** | Sciophilous; Habitat generalist
	*Argyronetaaquatica* (Clerck, 1757)	This is an aquatic spider that can be found in clean, vegetated freshwater where there is little current (Roberts 1996; Harvey et al. 2002b; Nentwig et al. 2012), for example, ponds, lakes, pools, calm rivers, ditches, canals (Locket and Millidge 1953; Harvey et al. 2002b; Almquist 2005). Vilbaste (1980) found this species in fens and bogs. *A.aquatica*, though air-breathing, is entirely aquatic – it is the only spider that spends its whole life under water (Locket and Millidge 1953; Bromhall 1988; Schütz and Taborsky 2003).	**Hygrophilous** | Xerophilous; Photophilous | Sciophilous; Habitat generalist *Sub-group*: Aquatic species
Eutichuridae	*Cheiracanthiumerraticum* (Walckenaer, 1802)	It inhabits open localities (Nentwig et al. 2012; Arachnologische Gesellschaft 2018). The main habitats of this species in central Europe are chalk grasslands and heathland (Bonte et al. 2003, 2004). It can also be found in fens and bogs (Vilbaste 1980; Arachnologische Gesellschaft 2018).	Hygrophilous | Xerophilous; **Photophilous** | Sciophilous; Habitat generalist
Eutichuridae	*Cheiracanthiumpunctorium* (Villers, 1789)	It can be found in warm, open habitats (Nentwig et al. 2012). It occurs, for example, in dry grasslands, damp clearings, wasteland, moist meadows, swamps (Roberts 1996; Komposch 2000; Almquist 2006; Arachnologische Gesellschaft 2018).	Hygrophilous | Xerophilous; **Photophilous** | Sciophilous; Habitat generalist
Gnaphosidae	*Drassodeslapidosus* (Walckenaer, 1802)	It is usually found in dry habitats with very sparse or no vegetation (Roberts 1996; Bonte et al. 2003, 2004; Arachnologische Gesellschaft 2018). It occurs in stony areas (e.g. scree), in dry grasslands, the drier parts of shingle beaches and elsewhere (Roberts 1996; Harvey et al. 2002b; Arachnologische Gesellschaft 2018). According to Nentwig et al. (2012), however, *D.lapidosus* can occur from very dry to swampy situations. This species was found in fens by Kajak et al. (2000). In addition, *D.lapidosus* is a synanthropic species – it is associated with human-influenced habitats and can be found, for example, in gardens, waste ground, industrial sites and in buildings (Harvey et al. 2002a; Nentwig et al. 2012; Arachnologische Gesellschaft 2018).	Hygrophilous | Xerophilous; **Photophilous** | Sciophilous; Habitat generalist *Sub-group*: Synanthropic species
	*Drassodespubescens* (Thorell, 1856)	It can be found in different situations – from dry to moist habitats (Matveinen-Huju et al. 2006; Nentwig et al. 2012), from grasslands and heathlands to open coniferous and deciduous forests (Locket and Millidge 1951; Harvey et al. 2002b; Almquist 2006; Nentwig et al. 2012; Arachnologische Gesellschaft 2018). *D.pubescens* has been found also in fens (Vilbaste 1980) and bogs (Koponen 2002a,b; Rėlys and Dapkus 2002; Rėlys et al. 2002).	Hygrophilous | Xerophilous; Photophilous | Sciophilous; **Habitat generalist**
	*Drassylluslutetianus* (L. Koch, 1866)	It has been recorded from different habitats, for example, moist meadows, water borders, sand dunes, sea shore, alluvial forests etc. (Harvey et al. 2002b; Almquist 2006; Nentwig et al. 2012). It has also been found in fens (Vilbaste 1980) and different bog habitats (Kupryjanowicz et al. 1998; Koponen 2002b, 2003; Rėlys et al. 2002; Oxbrough et al. 2006). Also, *D.lutetianus* occurs in disturbed habitats such as arable land and gardens (Cristofoli et al. 2010; Arachnologische Gesellschaft 2018).	Hygrophilous | Xerophilous; Photophilous | Sciophilous; **Habitat generalist**
	*Drassylluspraeficus* (L. Koch, 1866)	It can be found in dry and open habitats (Heimer and Nentwig 1991). It occurs in dry grasslands, sparse pine-woods, rocky steppes, shores and in similar habitats (Koponen 2000; Almquist 2006; Nentwig et al. 2012; Arachnologische Gesellschaft 2018). It is also sometimes found on dry heathland, mostly between about 6 to 12 years after fire (Harvey et al. 2002b).	Hygrophilous | **Xerophilous**; **Photophilous** | Sciophilous; Habitat generalist
	*Drassylluspusillus* (C. L. Koch, 1833)	It has a preference for dry situations (Locket and Millidge 1951; Roberts 1996; Arachnologische Gesellschaft 2018). It can be found in very different habitats – in chalk grasslands, heathlands, dry meadows, river-floodplains, stony pine and mixed forests etc. (Heimer and Nentwig 1991; Roberts 1996; Bonte et al. 2004; Almquist 2006; Nentwig et al. 2012). It has also been collected in bogs (Koponen 2002a,b; Rėlys et al. 2002).	Hygrophilous | **Xerophilous**; Photophilous | Sciophilous; Habitat generalist
	*Gnaphosabicolor* (Hahn, 1833)	A species that favors light forests and other open habitats (Matveinen-Huju et al. 2006; Nentwig et al. 2012). The species has been found, for example, in open pine forests (Pommeresche 2002; Almquist 2006), burnt forests (Moretti et al. 2002), rocky steppes (Heimer and Nentwig 1991; Nentwig et al. 2012), screes (Arachnologische Gesellschaft 2018) and heathlands (Almquist 2006).	Hygrophilous | **Xerophilous**; **Photophilous** | Sciophilous; Habitat generalist
	*Gnaphosalapponum* (L. Koch, 1866)	It is a bog-inhabitant, which is quite abundant in bogs of Northern Europe (Koponen 2002a,b, 2003; Almquist 2006). Interestingly, that Koponen (1991) observed that in southern Finland this species occurs only on bogs, while in the northernmost region of Finland it is markedly eurytopic, i.e. able to live in a wide variety of habitats.	**Hygrophilous** | Xerophilous; Photophilous | Sciophilous; Habitat generalist *Sub-group*: Bog species
Gnaphosidae	*Gnaphosanigerrima* L. Koch, 1877	It shows a clear preference for *Sphagnum* mosses (Harvey et al. 2002b; Boyce 2004; Platen 2004; Almquist 2006). It occurs in bogs and swampy places (Roberts 1996; Kupryjanowicz et al. 1998; Koponen 2002b; Rėlys and Dapkus 2002; Oliger 2004; Nentwig et al. 2012).	**Hygrophilous** | Xerophilous; Photophilous | Sciophilous; Habitat generalist *Sub-group*: Sphagnophilous species
	*Haplodrassusmoderatus* (Kulczyński, 1897)	It is often recorded from peatbogs and fenlands (Vilbaste 1980; Koponen 2002a; Rėlys and Dapkus 2002a; Rėlys et al. 2002). Overall, however, it has been found in a range of damp habitats – humid meadows, moist alder forests, swamps etc. (Almquist 2006; Nentwig et al. 2012).	**Hygrophilous** | Xerophilous; Photophilous | Sciophilous; Habitat generalist
	*Haplodrassussignifer* (C. L. Koch, 1839)	It has mainly be found in dry habitats (Bonte et al. 2003, 2004; Arachnologische Gesellschaft 2018) – on sand and stony places, heathlands (Harvey et al. 2002b; Nentwig et al. 2012), dry grasslands (Almquist 2006; Nentwig et al. 2012; Arachnologische Gesellschaft 2018), pine forests (Rėlys and Dapkus 2002; Biteniekyté and Rélys 2008). Although this species seems to prefer dry habitats, these can often be found in slightly raised, dry patches within otherwise wet and boggy areas (Roberts 1996). The species is also found in fens (Vilbaste 1980) and bogs (Vilbaste 1980; Kupryjanowicz et al. 1998; Koponen 2002a,b; Rėlys and Dapkus 2002; Rėlys et al. 2002; Bruun and Toft 2004; Biteniekyté and Rélys 2008).	Hygrophilous | **Xerophilous**; Photophilous | Sciophilous; Habitat generalist *Note*: Also within wet habitats on dry, raised patches of vegetation
	*Haplodrassussilvestris* (Blackwall, 1833)	It is a forest species (Locket and Millidge 1951; Roberts 1996; Harvey et al. 2002b; Nentwig et al. 2012) that can live in different types of forests, including both deciduous and pine forests (Almquist 2006; Arachnologische Gesellschaft 2018). Sometimes it can also be found in dry meadows and bogs (Rėlys et al. 2002; Nentwig et al. 2012).	Hygrophilous | Xerophilous; Photophilous | **Sciophilous**; Habitat generalist *Sub-group*: Forest generalist
	*Micariapulicaria* (Sundevall, 1831)	It has been recorded from a variety of situations which are open to sunshine (Heimer and Nentwig 1991; Roberts 1996), but particularly the warm, sunny parts of sandy heaths, chalk downlands, dunes and derelict land (Harvey et al. 2002b). This species has also been found in meadows close to lakes, saltmarshes, open pine forests, broad-leaved woodlands, fens, bogs, as well as in stony, bare and dry habitats (Vilbaste 1980; Harvey et al. 2002b; Rėlys et al. 2002; Almquist 2006; Matveinen-Huju et al. 2006).	Hygrophilous | Xerophilous; **Photophilous** | Sciophilous; Habitat generalist *Sub-group*: Photophilous generalist
	*Poecilochroavariana* (C. L. Koch, 1839)	It occurs in dry and sun exposed, stony or sandy habitats (Roberts 1996; Nentwig et al. 2012). It can be found in dry meadows, dune heaths and open pine woods (Almquist 2006).	Hygrophilous | **Xerophilous**; **Photophilous** | Sciophilous; Habitat generalist
	*Zelotesclivicola* (L. Koch, 1870)	This species is associated mainly with open forests (Almquist 2006; Matveinen-Huju et al. 2006; Nentwig et al. 2012). It can be found in pine forests (Pommeresche 2002; Rėlys and Dapkus 2002b), beech woodland, mixed deciduous and coniferous woodland and thermophile woodland fringes (Arachnologische Gesellschaft 2018). This species can be found also in other dry habitats such as heaths (Cattin et al. 2003; Arachnologische Gesellschaft 2018). Also, it can be found in bogs (Kupryjanowicz et al. 1998; Rėlys and Dapkus 2002b; Rėlys et al. 2002).	Hygrophilous | **Xerophilous**; **Photophilous** | Sciophilous; Habitat generalist
	*Zeloteslatreillei* (Simon, 1878)	It prefers open habitats (Harvey et al. 2002b; Oxbrough et al. 2006) and is usually found in dry habitats – in chalk grasslands, heathlands (Gajdoš and Toft 2000; Harvey et al. 2002b; Bonte et al. 2003, 2004), coastal dunes and sandy shores (Arachnologische Gesellschaft 2018), dry pine forests (Rėlys and Dapkus 2002b; Almquist 2006). This species can also be found in dry, raised patches of vegetation within marshy sites (Roberts 1996). Has been recorded from fens (Kajak et al. 2000) and bogs (Koponen 2002a, 2003; Rėlys and Dapkus 2002; Rėlys et al. 2002).	Hygrophilous | **Xerophilous**; **Photophilous** | Sciophilous; Habitat generalist *Note*: Also within wet habitats on dry, raised patches of vegetation
Gnaphosidae	*Zelotessubterraneus* (C. L. Koch, 1833)	This species is able to live in a variety of habitat types – in woods, heathland, boggy areas, dry meadows, screes, stony areas etc. (Almquist 2006; Nentwig et al. 2012; Arachnologische Gesellschaft 2018). The preference of this species, however, seems to be on forests (Rėlys and Dapkus 2002b; Arachnologische Gesellschaft 2018). Also, it can be found in coastal habitats (Harvey et al. 2002b; Bonte et al. 2003; Arachnologische Gesellschaft 2018).	Hygrophilous | Xerophilous; Photophilous | Sciophilous; **Habitat generalist**
Hahniidae	*Antisteaelegans* (Blackwall, 1841)	It has been recorded from a variety of damp, open habitats, for example, bogs, poor fens, wet heathlands, moist pastures and others (Harvey et al. 2002b; Almquist 2005; Oxbrough et al. 2007; Cristofoli et al. 2010). It seems that *A.elegans* is especially abundant in fens and bogs – Kajak et al. (2000) found it among the dominant spider species in natural fens in Poland; Koponen (2002b, 2003) found this species dominating in peatbogs of Finland; and Rėlys et al. (2002) wrote that *A.elegans* is typically abundant peatbog species in Lithuania. *A.elegans* has been recorded also in other studies where fens and bogs have been investigated (e.g., Vilbaste 1980; Kupryjanowicz et al. 1998; Koponen 2002a).	**Hygrophilous** | Xerophilous; **Photophilous** | Sciophilous; Habitat generalist
Linyphiidae	*Agynetamollis* (O. Pickard-Cambridge, 1871)	It is associated with damp conditions (Harvey et al. 2002a; Oxbrough et al. 2007). It lives mainly in grasslands (Harvey et al. 2002a; Arachnologische Gesellschaft 2018), but can be found also in woods (Locket and Millidge 1953). Occurs also in mires, including fens and bogs (Vilbaste 1980; Oxbrough et al. 2006).	**Hygrophilous** | Xerophilous; Photophilous | Sciophilous; Habitat generalist
	*Agynetasubtilis* (O. Pickard-Cambridge, 1863)	This species is a forest generalist (Matveinen-Huju et al. 2006) – it has been found in different types of forest habitats, including broad-leaved woodland (Harvey et al. 2002a), pine forest (Rėlys and Dapkus 2002b), *Sphagnum* birch forest (Arachnologische Gesellschaft 2018). Can be found also in other habitats, for example, meadows, bogs, coastal and heathland habitats (Rėlys and Dapkus 2002b; Nentwig et al. 2012; Arachnologische Gesellschaft 2018). This species is indifferent as regards soil moisture (Matveinen-Huju et al. 2006).	Hygrophilous | Xerophilous; Photophilous | **Sciophilous**; Habitat generalist *Sub-group*: Forest generalist
	*Allomengeavidua* (L. Koch, 1879)	It is found in a variety of usually very damp and flooded habitats, e.g., different swamps and marshes (Harvey et al. 2002a; Oxbrough et al. 2006; Nentwig et al. 2012). Kajak et al. (2000) has found this species in fens of Poland.	**Hygrophilous** | Xerophilous; Photophilous | Sciophilous; Habitat generalist
	*Bathyphantesgracilis* (Blackwall, 1841)	A typical species for moist habitats (Koponen 2002b; Matveinen-Huju et al. 2006). It can be found in grasslands, heathlands, forests (Harvey et al. 2002a; Nentwig et al. 2012), as well as in fens (Vilbaste 1980; Kajak et al. 2000) and bogs (Koponen 2002a,b; Rėlys et al. 2002). Also, *B.gracilis* is an agrobiont – it is very common in open agricultural habitats, for example, meadows and fields (Bonte et al. 2002; Pommeresche 2004; Cristofoli et al. 2010). The species is a common aeronaut (Locket and Millidge 1953; Bonte et al. 2002; Harvey et al. 2002a).	**Hygrophilous** | Xerophilous; Photophilous | Sciophilous; Habitat generalist *Sub-group*: Agrobiontic species
	*Bathyphantesnigrinus* (Westring, 1851)	It is a hygrophilous species (Aakra 2002; Matveinen-Huju et al. 2006) which seems to have an affinity for forests (Harvey et al. 2002a; Cristofoli et al. 2010). It mainly occurs in very damp and shadowed places, especially in bog forests (Nentwig et al. 2012). In Latvia it has been found on fens by Cera et al. (2010).	**Hygrophilous** | Xerophilous; Photophilous | **Sciophilous**; Habitat generalist
	*Bathyphantesparvulus* (Westring, 1851)	It is predominantly a grassland spider that occurs in acid grasslands, chalk grasslands and meadows (Harvey et al. 2002a; Cristofoli et al. 2010). This species, however, can also be found on fens (Kajak et al. 2000), bogs (Koponen 2002a) and forests (Heimer and Nentwig 1991; Nentwig et al. 2012).	Hygrophilous | Xerophilous; **Photophilous** | Sciophilous; Habitat generalist *Sub-group*: Grassland species
	*Bolyphanthesalticeps* (Sundevall, 1833)	It is indifferent as regards light intensity (Matveinen-Huju et al. 2006) and can be found in a variety of habitats – in grasslands, forest edges, coniferous and broad-leaved woodlands (Harvey et al. 2002a; Nentwig et al. 2012). It can also occur in fens and bogs (Vilbaste 1980; Arachnologische Gesellschaft 2018).	Hygrophilous | Xerophilous; Photophilous | Sciophilous; **Habitat generalist**
	*Centromerussemiater* (L. Koch, 1879)	It can be found in a wide range of wet habitats, for example, in bogs, fens, reed-beds, humid meadows etc. (Kajak et al. 2000; Stańska et al. 2000; Harvey et al. 2002a). Also, the species can be detected in coastal habitats (Arachnologische Gesellschaft 2018).	**Hygrophilous** | Xerophilous; Photophilous | Sciophilous; Habitat generalist *Sub-group*: Hygrophilous generalist
	*Ceratinellabrevipes* (Westring, 1851)	It might be found in various habitats, including seasonally wet and wet grasslands (Arachnologische Gesellschaft 2018), wet woodland with *Sphagnum* (Glime and Lissner 2013), reed-beds (Harvey et al. 2002a), open agricultural habitats (Cristofoli et al. 2010), as well as in other situations (Harvey et al. 2002a; Nentwig et al. 2012; Arachnologische Gesellschaft 2018). Kajak et al. (2000) found the species in fens, but Vilbaste (1980) – in fens and bogs.	**Hygrophilous** | Xerophilous; Photophilous | Sciophilous; Habitat generalist *Sub-group*: Hygrophilous generalist
Linyphiidae	*Diplostylaconcolor* (Wider, 1834)	It can be found in a wide variety of situations – in grasslands, hedgerows, gardens, humid forests, marshes and shadowed watersides (Harvey et al. 2002a; Nentwig et al. 2012). Overall, *D.concolor* seems to prefer forest habitats (Stańska et al. 2000; Pommeresche 2002, 2004; Buchholz 2009; Gallé et al. 2011). Also, this species is quite common in habitats with a high level of human disturbance, such as vineyards (Harvey et al. 2002a; Isaia et al. 2007; Arachnologische Gesellschaft 2018).	**Hygrophilous** | Xerophilous; Photophilous | Sciophilous; Habitat generalist
	*Dismodicuselevatus* (C. L. Koch, 1838)	A species that is related with trees, particularly with conifers – it occurs in pine forests and in fir and spruce woodlands (Matveinen-Huju et al. 2006; Arachnologische Gesellschaft 2018). *D.elevatus* can be found mostly under pines, on the lower branches of pines, and also on heather, gorse and juniper (Locket and Millidge 1953; Harvey et al. 2002a; Nentwig et al. 2012). Can be also found in fens and bogs (Vilbaste 1980; Rėlys et al. 2002; Cera et al. 2010).	Hygrophilous | Xerophilous; Photophilous | **Sciophilous**; Habitat generalist *Sub-group*: Coniferous forest species
	*Erigonearctica* (White, 1852)	It prefers humid conditions (Nentwig et al. 2012). This species is mainly associated with coastal habitats (Hänggi et al. 1995) – it occurs on the seashore and the shoreline of estuaries where it can be found amongst stones and seaweed (Locket and Millidge 1953; Harvey et al. 2002a). Irmler et al. (2002) have discovered *E.arctica* in the coastal salt marsh. Inland this species can also be found in saline areas (Harvey et al. 2002a; Duffey 2005).	**Hygrophilous** | Xerophilous; Photophilous | Sciophilous; Habitat generalist *Sub-groups*: Coastal species, Halophilous species
	*Erigoneatra* Blackwall, 1833 *& Erigonedentipalpis* (Wider, 1834)	Both these spiders can be classified as pioneer species (Aakra 2002). *E.atra* is an universally distributed species – it is one of the commonest spiders that often disperse aeronautically in large numbers (Locket and Millidge 1953; Harvey et al. 2002a). The second species – *E.dentipalpis* – occurs in a similarly wide range of habitats as *E.atra*, and is an equally common aeronaut (Locket and Millidge 1953; Harvey et al. 2002a). Both these linyphiids have also been described as ruderal species – they show a high frequency of occurrence in ruderal sites, fields and gardens, i.e., sites of agricultural disturbance (Bonte et al. 2002; Cole et al. 2003). In addition, both of these linyphiids have been found among the most abundant species in different European agroecosystems, and thus are also called agrobiont species (Thomas and Jepson 1997; Feber et al. 1998; Ratschker and Roth 2000; Pommeresche 2004; Thorbek and Bilde 2004; Schmidt and Tscharntke 2005; Öberg et al. 2007; Pommeresche et al. 2013).	Hygrophilous | Xerophilous; Photophilous | Sciophilous; **Habitat generalist***Sub-group*: Agrobiontic species
	*Erigonellahiemalis* (Blackwall, 1841)	It has been recorded in a wide variety of habitats (Harvey et al. 2002a; Nentwig et al. 2012; Arachnologische Gesellschaft 2018), but perhaps its main habitat is forest (Locket and Millidge 1953; Harvey et al. 2002a; Oxbrough et al. 2006; Nentwig et al. 2012). The species can also occur in bogs (Vilbaste 1980). According to Matveinen-Huju et al. (2006), *E.hiemalis* is indifferent as regards soil moisture.	Hygrophilous | Xerophilous; Photophilous | Sciophilous; **Habitat generalist**
	*Erigonellaignobilis* (O. Pickard-Cambridge, 1871)	It usually occurs in damp, swampy habitats, damp litter and low vegetation at the edge of open water (Locket and Millidge 1953; Harvey et al. 2002a; Oxbrough et al. 2006; Nentwig et al. 2012). It has been found in Atlantic hay meadows, seasonally wet and wet grasslands (Arachnologische Gesellschaft 2018), as well as in mires (Vilbaste 1980; Arachnologische Gesellschaft 2018).	**Hygrophilous** | Xerophilous; Photophilous | Sciophilous; Habitat generalist
	*Floroniabucculenta* (Clerck, 1757)	It occurs in damp places in a variety of habitats (Harvey et al. 2002a). It can be found in mires, reed-beds, grasslands, open woodland, on earthy banks, in damp forest edges and elsewhere (Locket and Millidge 1953; Harvey et al. 2002a; Rėlys and Dapkus 2002; Nentwig et al. 2012; Arachnologische Gesellschaft 2018).	**Hygrophilous** | Xerophilous; Photophilous | Sciophilous; Habitat generalist *Sub-group*: Hygrophilous generalist
	*Gnathonariumdentatum* (Wider, 1834)	A strictly hygrophilous species – it is usually found near water (Hänggi et al. 1995; Nentwig et al. 2012). It occurs, for example, by the side of streams (Locket and Millidge 1953), in reed swamps (Duffey 2005) and in other flooded habitats (Harvey et al. 2002a; Cattin et al. 2003). Vilbaste (1980) has found this species in fens and bogs of Estonia.	**Hygrophilous** | Xerophilous; Photophilous | Sciophilous; Habitat generalist
Linyphiidae	*Gongylidiellumlatebricola* (O. Pickard-Cambridge, 1871)	It can be found in wet habitats (Oxbrough et al. 2006). It occurs in damp situations in woodland, grasslands, and bogs (Harvey et al. 2002a). The main habitats of this species seems to be different forests, including fir and spruce woodlands, *Sphagnum* birch woods, beech woodland, pine forests etc. (Locket and Millidge 1953; Matveinen-Huju et al. 2006; Nentwig et al. 2012; Arachnologische Gesellschaft 2018).	**Hygrophilous** | Xerophilous; Photophilous | **Sciophilous**; Habitat generalist
	*Kaestneriapullata* (O. Pickard-Cambridge, 1863)	It is common in wet habitats such as marshlands, reed-beds, seeps, drainage ditches, wet grasslands etc. (Harvey et al. 2002a; Oxbrough et al. 2006; Arachnologische Gesellschaft 2018). Can be also found in fens (Vilbaste 1980; Kajak et al. 2000; Cera et al. 2010) and bogs (Vilbaste 1980).	**Hygrophilous** | Xerophilous; Photophilous | Sciophilous; Habitat generalist *Sub-group*: Hygrophilous generalist
	*Linyphiahortensis* Sundevall, 1830	It can be found in various habitats (Nentwig et al. 2012; Arachnologische Gesellschaft 2018), however, it is mostly found in woods (Locket and Millidge 1953; Roberts 1996; Harvey et al. 2002a; Arachnologische Gesellschaft 2018). It occurs, for example, in broadleaved deciduous woodlands, mixed deciduous-coniferous woodlands, mixed fir-spruce-beech woodlands and in other forest types (Arachnologische Gesellschaft 2018).	Hygrophilous | Xerophilous; Photophilous | **Sciophilous**; Habitat generalist
	*Micrargusherbigradus* (Blackwall, 1854)	It is usually found in forests (Locket and Millidge 1953; Harvey et al. 2002a; Nentwig et al. 2012; Arachnologische Gesellschaft 2018). It occurs in different forest types – in beech woodlands, fir and spruce woodlands, *Sphagnum* birch woods, broadleaved deciduous woodlands and others (Arachnologische Gesellschaft 2018). This species, however, inhabits also bogs – Šternbergs (1991) has found it in a Baži bog in Latvia, while Spuņģis (2008) has caught it in several different bogs of Latvia. Vilbaste (1980) has found *M.herbigradus* in bogs of Estonia, but Rėlys and Dapkus (2002) collected this species in a pine bog and the surrounding pine forest in Lithuania.	Hygrophilous | Xerophilous; Photophilous | **Sciophilous**; Habitat generalist *Sub-group*: Forest generalist
	*Microlinyphiaimpigra* (O. Pickard-Cambridge, 1871)	It inhabits marshy habitats (Roberts 1996; Harvey et al. 2002a; Nentwig et al. 2012). It can be found in the littoral zone of inland surface waterbodies, reed-beds and mires (Arachnologische Gesellschaft 2018). Koponen (2000) has found this species on sandy shores (Koponen 2000).	**Hygrophilous** | Xerophilous; Photophilous | Sciophilous; Habitat generalist
	*Microlinyphiapusilla* (Sundevall, 1830)	It has an affinity for moist open habitats (Heimer and Nentwig 1991; Nentwig et al. 2012). It can be found in heathland, dune, saltmarsh and other wet habitats, but is perhaps commonest in grasslands (Harvey et al. 2002a; Arachnologische Gesellschaft 2018). It has been found also in bogs (Vilbaste 1980) and fens (Vilbaste 1980; Kajak et al. 2000).	**Hygrophilous** | Xerophilous; **Photophilous** | Sciophilous; Habitat generalist
	*Nerienemontana* (Clerck, 1757)	It can be found on bushes and low vegetation and on tree trunks, logs and a variety of other structures in a range of habitats (Roberts 1996). This species, however, occurs mainly in woodland and other shady places (Harvey et al. 2002a; Oxbrough et al. 2006). It can be found also on bogs (Vilbaste 1980).	Hygrophilous | Xerophilous; Photophilous | **Sciophilous**; Habitat generalist
	*Notioscopussarcinatus* (O. Pickard-Cambridge, 1873)	It prefers humid conditions (Locket and Millidge 1953; Nentwig et al. 2012). It occurs in wet, marshy areas, especially in different kinds of mires, including fens (Vilbaste 1980; Kajak et al. 2000; Boyce 2004) and bogs (Vilbaste 1980; Šternbergs 1991; Kupryjanowicz et al. 1998; Pommeresche 2002; Rėlys and Dapkus 2002). The species is mainly found in tall moss (*Sphagnum*, *Polytrichum*), often under pine or other trees in the swampy places (Harvey et al. 2002a; Nentwig et al. 2012). *N.sarcinatus* can also be found in moist grasslands (Hänggi et al. 1995; Arachnologische Gesellschaft 2018).	**Hygrophilous** | Xerophilous; Photophilous | Sciophilous; Habitat generalist
	*Pocadicnemispumila* (Blackwall, 1841)	It occurs in a variety of situations, including grasslands, heathlands, forests, marshes (Locket and Millidge 1953; Harvey et al. 2002a; Biteniekyté, Rélys 2008; Nentwig et al. 2012). Overall, however, it prefers moist habitats (Heimer and Nentwig 1991; Nentwig et al. 2012). It seems to be a typical species in bogs (Vilbaste 1980; Kupryjanowicz et al. 1998; Rėlys and Dapkus 2002; Rėlys et al. 2002; Koponen 2003), and can be found also in fens (Vilbaste 1980; Kajak et al. 2000).	**Hygrophilous** | Xerophilous; Photophilous | Sciophilous; Habitat generalist
Linyphiidae	*Styloctetorcompar* (Westring, 1861)	According to Nentwig et al. (2012), the species needs humid conditions. *S.compar* is mainly a grassland spider (Arachnologische Gesellschaft 2018), but it can also be found in peatbogs and weatlands (Miller 1971).	**Hygrophilous** | Xerophilous; Photophilous | Sciophilous; Habitat generalist
	*Tallusiaexperta* (O. Pickard-Cambridge, 1871)	A wetland species which inhabits a variety of wet marshy habitats (Harvey et al. 2002a; Oxbrough et al. 2006), including bogs, fens and reed-beds (Harvey et al. 2002a; Koponen 2002a; Rėlys and Dapkus 2002; Rėlys et al. 2002). *T.experta* can also be found in wet meadows and forest edges (Heimer and Nentwig 1991; Nentwig et al. 2012).	**Hygrophilous** | Xerophilous; Photophilous | Sciophilous; Habitat generalist *Sub-group*: Hygrophilous generalist
	*Tenuiphantescristatus* (Menge, 1866)	It can be found in a variety of damp, forested habitats (Harvey et al. 2002a; Matveinen-Huju et al. 2006). It occurs, for example, in beech woodland, broad-leaved swamp woodland on acid peat, birch and pine on *Sphagnum*, juniper scrub on limestone etc. (Harvey et al. 2002a; Arachnologische Gesellschaft 2018). According to Heimer and Nentwig (1991) and Nentwig et al. (2012), *T.cristatus* lives mainly in deciduous forests. It can also be found on bogs (Rėlys et al. 2002; Spuņģis 2008).	**Hygrophilous** | Xerophilous; Photophilous | **Sciophilous**; Habitat generalist
	*Trichopternoidesthorelli* (Westring, 1861)	It is associated with wet conditions but it is not bound to any particular habitat (Oxbrough et al. 2007; Nentwig et al. 2012). It can be found, for example, in wet heathlands, fens and bogs (Vilbaste 1980; Harvey et al. 2002a).	**Hygrophilous** | Xerophilous; Photophilous | Sciophilous; Habitat generalist *Sub-group*: Hygrophilous generalist
	*Typhochrestusdigitatus* (O. Pickard-Cambridge, 1873)	It inhabits dry and warm locations, for example, sandhills, heathlands, grasslands and other bare or sparsely vegetated habitats (Locket and Millidge 1953; Harvey et al. 2002a; Nentwig et al. 2012; Arachnologische Gesellschaft 2018). This species seems to have a distinct preference for coastal habitats (e.g., grey dunes, coastal grasslands), at least in central Europe (Hänggi et al. 1995; Bonte et al. 2003, 2004).	Hygrophilous | **Xerophilous**; **Photophilous** | Sciophilous; Habitat generalist *Sub-group*: Coastal species
	*Walckenaeriaalticeps* (Denis, 1952)	It inhabits different types of forest habitats – it has been recorded from beech woodland, *Sphagnum* birch woods, fir and spruce forest, pine forest (Rėlys and Dapkus 2002; Biteniekyté and Rélys 2008; Arachnologische Gesellschaft 2018), as well as from forest edges (Duffey 2005). Also, this species is usually found in *Sphagnum* bogs, including both open bogs and pine bogs (Kupryjanowicz et al. 1998; Harvey et al. 2002b; Rėlys and Dapkus 2002; Rėlys et al. 2002; Biteniekyté and Rélys 2008), as well as in other sites with moist and shaded *Sphagnum* (Harvey et al. 2002b; Nentwig et al. 2012). The species can be found also in coastal dunes and sandy shores (Arachnologische Gesellschaft 2018).	**Hygrophilous** | Xerophilous; Photophilous | **Sciophilous**; Habitat generalist
	*Walckenaeriaatrotibialis* (O. Pickard-Cambridge, 1878)	It occurs in various moist habitats (Harvey et al. 2002a; Matveinen-Huju et al. 2006). This species is indifferent as regards light intensity (Matveinen-Huju et al. 2006) – it has been found in different open habitats such as grasslands, fens, bogs (Kupryjanowicz et al. 1998; Harvey et al. 2002a; Koponen 2002b; Rėlys et al. 2002; Spuņģis 2008), as well as in shaded habitats (Stańska et al. 2000; Buchholz 2010; Arachnologische Gesellschaft 2018).	**Hygrophilous** | Xerophilous; Photophilous | Sciophilous; Habitat generalist
	*Walckenaeriavigilax* (Blackwall, 1853)	It occurs in wet habitats (Harvey et al. 2002a; Matveinen-Huju et al. 2006; Oxbrough et al. 2006; Nentwig et al. 2012). It can be found in grasslands, saltmarshes, arable land, gardens and in other places (Harvey et al. 2002a; Arachnologische Gesellschaft 2018). In Norway, *W.vigilax* is a typical riparian species which is restricted to river banks (Aakra 2002).	**Hygrophilous** | Xerophilous; Photophilous | Sciophilous; Habitat generalist *Sub-group*: Riparian species (in Norway)
Liocranidae	*Agroecadentigera* Kulczyński, 1913	In the continental Europe this species can be found in a variety of damp habitats, especially on mires (Kajak et al. 2000; Harvey et al. 2002b; Koponen 2002b; Rėlys and Dapkus 2002a; Rėlys et al. 2002), while in the United Kingdom this species occurs in coastal sand dunes (Harvey et al. 2002b).	**Hygrophilous** | Xerophilous; Photophilous | Sciophilous; Habitat generalist *Note*: Preferred habitats differ geographically
Liocranidae	*Agroecaproxima* (O. Pickard-Cambridge, 1871)	It has a preference for fairly dry habitats (Roberts 1996; Harvey et al. 2002b). It is one of the commonest species on heathland (Harvey et al. 2002b). It is also a characteristic species of coastal dunes and sandy shores (Almquist 2006; Finch et al. 2007; Arachnologische Gesellschaft 2018). Also, it can be found in woodland clearings, dry pine woods (Roberts 1996; Almquist 2006) and on bogs (Kupryjanowicz et al. 1998; Koponen 2002a,b; Rėlys and Dapkus 2002; Rėlys et al. 2002; Biteniekyté, Rélys 2008).	**Hygrophilous** | Xerophilous; Photophilous | Sciophilous; Habitat generalist *Sub-group*: Heathland species
	*Liocranoecastriata* (Kulczyński, 1882)	It occurs in different moist places with no clear preference for any particular type of wet habitat (Harvey et al. 2002b). It can be found in habitats such as bogs, fens, wet heathlands, wet grasslands, damp woodland sites, forest meadows, stony shores and other similar habitats (Roberts 1996; Kajak et al. 2000; Harvey et al. 2002b; Almquist 2006).	**Hygrophilous** | Xerophilous; Photophilous | Sciophilous; Habitat generalist *Sub-group*: Hygrophilous generalist
	*Scotinapalliardi* (L. Koch, 1881)	The data of Kupryjanowicz et al. (1998) showed that this species does not occur outside raised peat bogs, so they suggested to classify it as a tyrphobiont (= species that inhabits only bogs). Indeed, many studies confirm that *S.palliardi* is very frequent in bogs (Vilbaste 1980; Šternbergs 1991; Kupryjanowicz et al. 1998 Rėlys and Dapkus 2002; Koponen 2002a,b, 2003; Rėlys et al. 2002; Rėlys and Dapkus 2002a,b; Koponen 2003; Biteniekyté and Rėlys 2008; Spuņģis 2008). Nevertheless, this species can also be found in chalk grasslands and heathlands, at least in central Europe (Roberts 1996; Bonte et al. 2003, 2004; Almquist 2006; Nentwig et al. 2012; Arachnologische Gesellschaft 2018).	**Hygrophilous** | Xerophilous; Photophilous | Sciophilous; Habitat generalist *Sub-group*: Bog species
Lycosidae	*Alopecosapulverulenta* (Clerck, 1757)	It has been found in many different open habitat types such as meadows, pastures, heathland, moorland, dunes, open forests, old quarries, urban gardens and cultivated land (Locket and Millidge 1951; Roberts 1996; Harvey et al. 2002b; Almquist 2005; Nentwig et al. 2012). It has also been frequently reported from peat bogs (Vilbaste 1980; Šternbergs 1991; Kupryjanowicz et al. 1998; Koponen 2002a,b; Rėlys and Dapkus 2002; Rėlys et al. 2002; Spuņģis 2008). Kajak et al. (2000) have found this species in fens.	Hygrophilous | Xerophilous; **Photophilous** | Sciophilous; Habitat generalist *Sub-group*: Photophilous generalist
	*Arctosaleopardus* (Sundevall, 1833)	It favors wet, open habitats (Roberts 1996; Oxbrough et al. 2006). It occurs in wet heathlands, dune slacks (Harvey et al. 2002b), sand dunes, stony shores (Almquist 2005), open agricultural habitats (Cristofoli et al. 2010), reed belts, humid grasslands (Buchholz and Schröder 2013), fens (Kajak et al. 2000) and bogs (Vilbaste 1980). At the same time *A.leopardus* seems to be a halophilous species – in a couple of studies it was associated with salty habitats (Finch et al. 2007; Buchholz 2009).	**Hygrophilous** | Xerophilous; **Photophilous** | Sciophilous; Habitat generalist *Sub-group*: Halophilous species
	*Auloniaalbimana* (Walckenaer, 1805)	It usually prefers sunny and dry habitats (Nentwig et al. 2012; Arachnologische Gesellschaft 2018). It inhabits chalk grasslands, heathlands, sparse and rocky pine-woods, quarries (Harvey et al. 2002b; Bonte et al. 2004; Almquist 2005). It can also be found in bogs (Rėlys and Dapkus 2002; Rėlys et al. 2002; Štambuk and Erben 2002; Koponen 2003).	Hygrophilous | **Xerophilous**; **Photophilous** | Sciophilous; Habitat generalist
	*Hygrolycosarubrofasciata* (Ohlert, 1865)	It is found in damp habitats (Harvey et al. 2002b). It mainly occurs in wet forests and in fens (Locket and Millidge 1951; Vilbaste 1980; Roberts 1996; Kajak et al. 2000; Harvey et al. 2002b; Nentwig et al. 2012). According to Štambuk and Erben (2002), *H.rubrofasciata* is an alder forest species. This species can also be found in damp meadows (Almquist 2005) and on bogs (Vilbaste 1980; Kupryjanowicz et al. 1998; Koponen 2002b; Štambuk and Erben 2002; Rėlys et al. 2002; Biteniekyté and Rélys 2008).	**Hygrophilous** | Xerophilous; Photophilous | **Sciophilous**; Habitat generalist *Sub-group*: Alder forest species
	*Pardosafulvipes* (Collett, 1876)	It is mainly associated with grasslands (Holm and Kronestedt 1970; Roberts 1996; Almquist 2005) and arable land (Holm and Kronestedt 1970; Huhta and Raatikainen 1974; Almquist 2005). This species can be found also on wetlands, including fens and bogs (Vilbaste 1980; Komposch 2000).	Hygrophilous | Xerophilous; **Photophilous** | Sciophilous; Habitat generalist
Lycosidae	*Pardosalugubris* (Walckenaer, 1802)	It is a very common species in woods, especially on forest edges and in woodland clearings (Roberts 1996; Almquist 2005). It never seems to occur very far from woods (Locket and Millidge 1951) and can be found in the habitats edging forests (Aakra 2002; Biteniekyté and Rélys 2008). Koponen (2005) has recorded this species at the burned forest. *P.lugubris* occurs on mires as well – it has been found in fens (Kajak et al. 2000) and bogs (Vilbaste 1980; Kupryjanowicz et al. 1998; Rėlys et al. 2002).	Hygrophilous | Xerophilous; **Photophilous** | Sciophilous; Habitat generalist *Sub-group*: Ecotonal forest species
	*Pardosaprativaga* (L. Koch, 1870) & *Pardosapullata* (Clerck, 1757)	Both these species are often found together (Locket and Millidge 1951; Roberts 1996), however, *P.prativaga* is not so common as *P.pullata* which is one of the commonest species of the genus (Locket and Millidge 1951; Roberts 1996). Both species occur in a wide variety of open habitats, including grasslands, heathlands, woodland clearings, dunes, old quarries and roadside verges, as well as in wet places such as dyke edges, damp meadows, water borders and swampy areas (Locket and Millidge 1951; Harvey et al. 2002b; Almquist 2005; Nentwig et al. 2012; Arachnologische Gesellschaft 2018). Both of these species have been found in fens (Vilbaste 1980; Kajak et al. 2000) and bogs (Vilbaste 1980; Kupryjanowicz et al. 1998; Rėlys et al. 2002). *P.pullata* has been found in bogs also by Koponen (2002a,b) and Spuņģis (2008).	**Hygrophilous** | Xerophilous; **Photophilous** | Sciophilous; Habitat generalist
	*Pardosaproxima* (C. L. Koch, 1847)	It can be found in a variety of sparsely vegetated habitats but usually in moist and marshy places (Locket and Millidge 1951; Harvey et al. 2002b). This species is most likely to be found at coastal sites including earthy cliffs, saltmarsh, dune slacks and in streamside habitats (Harvey et al. 2002b). It often occurs also in grasslands and fields, in damp situations (Roberts 1996; Nentwig et al. 2012). In addition, *P.proxima* seems to be associated with habitats of anthropogenic disturbances, for example, gardens and arable land (Bonte et al. 2002; Harvey et al. 2002b; Arachnologische Gesellschaft 2018).	**Hygrophilous** | Xerophilous; **Photophilous** | Sciophilous; Habitat generalist
	*Pardosasaltans* Töpfer-Hofmann, 2000	It occurs mainly in forests (Bonte et al. 2002; Harvey et al. 2002b; Nentwig et al. 2012; Barsoum et al. 2014). It prefers broadleaved deciduous woodland, but can also occur in coniferous woodland (Arachnologische Gesellschaft 2018). The species might also be found in other habitats, for example, in anthropogenic herb stands, hedgerows, vineyards etc. (Arachnologische Gesellschaft 2018).	Hygrophilous | Xerophilous; Photophilous | **Sciophilous**; Habitat generalist
	*Pardosasphagnicola* (Dahl, 1908)	It is found in damp or marshy habitats and is related with *Sphagnum* mosses (Roberts 1996; Almquist 2005; Matveinen-Huju et al. 2006; Glime and Lissner 2013). This species can be classified as tyrphobiontic species according to Peus (1928). *P.sphagnicola* has been found in peat bogs by numerous authors, and it is usually among the most common and abundant species in different bog habitats in Europe (Vilbaste 1980; Kupryjanowicz et al. 1998; Koponen 2002a,b, 2003; Rėlys et al. 2002; Rėlys and Dapkus 2002; Spuņģis 2008). In addition, some authors have reported this species also from fens (Vilbaste 1980; Kajak et al. 2000).	**Hygrophilous** | Xerophilous; Photophilous | Sciophilous; Habitat generalist *Sub-groups*: Bog species, Sphagnophilous species
	*Piratapiraticus* (Clerck, 1757)	It is a strictly hygrophilous species (Hänggi et al. 1995). It lives near standing or slowly flowing water (Heimer and Nentwig 1991; Cattin et al. 2003; Nentwig et al. 2012). This species might be found in a variety of wet, marshy areas such as pond and stream margins (Harvey et al. 2002b; Graham et al. 2003), fens (Vilbaste 1980; Kajak et al. 2000), bogs (Vilbaste 1980; Koponen 2002a; Pommeresche 2002; Rėlys et al. 2002; Spuņģis 2008) and in other habitats (Arachnologische Gesellschaft 2018). Graham et al. (2003) defined *P.piraticus* as a semi-aquatic spider, since it was strongly associated with moist substrates and was active in the upper littoral zone of a pond.	**Hygrophilous** | Xerophilous; Photophilous | Sciophilous; Habitat generalist *Sub-group*: Semi aquatic species
Lycosidae	*Piratapiscatorius* (Clerck, 1757)	It is always found in very damp areas (Harvey et al. 2002b; Glime and Lissner 2013), most often near standing or slowly flowing water (Harvey et al. 2002b; Nentwig et al. 2012). Peus (1928) has classified *P.piscatorius* as a tyrphobiontic species. Indeed, this species is more typical for bog habitats (Koponen 2002a,b; Bruun, Toft 2004; Oliger 2004), however it can also be found in other wetlands as well, including fens (Vilbaste 1980; Kajak et al. 2000; Glime and Lissner 2013). This species shows a clear preferences for *Sphagnum* mosses – in bogs it is usually confined to the *Sphagnum* area of the habitat (Bruun, Toft 2004), and, in addition, the species can also be found in *Sphagnum* birch woods (Arachnologische Gesellschaft 2018).	**Hygrophilous** | Xerophilous; Photophilous | Sciophilous; Habitat generalist *Sub-groups*: Bog species; Sphagnophilous species
	*Piratatenuitarsis* Simon, 1876	It is mainly found in *Sphagnum* bogs often in the vicinity of bog pools (Roberts 1996; Harvey et al. 2002b). This species can be found also on fens, in wet heathlands, reed-beds, grasslands, woodland fringes and clearings (Kajak et al. 2000; Komposch 2000; Harvey et al. 2002b; Arachnologische Gesellschaft 2018).	**Hygrophilous** | Xerophilous; Photophilous | Sciophilous; Habitat generalist *Sub-group*: Bog species
	*Piratauliginosus* (Thorell, 1856)	According to Casemir (1976) this species is a true tyrphobiont. The recent evidence, however, shows that although this species is characteristic of bogs, it is not confined to them and thus is not a strict tyrphobiont but rather a tyrphophilous species (Hänggi et al. 1995; Neet 1996; Buchholz 2016). In any case, *P.uliginosus* is usually one of the characteristic and often most abundant species on European peat bogs (e.g., Kupryjanowicz et al. 1998; Koponen 2002a,b, 2003; Rėlys et al. 2002; Rėlys and Dapkus 2002). This species can also be found in fens, grasslands, heathland, woods (Kajak et al. 2000; Harvey et al. 2002b; Almquist 2005; Arachnologische Gesellschaft 2018). Overall, *P.uliginosus* prefers damp, open habitats (Štambuk and Erben 2002; Oxbrough et al. 2006; Nentwig et al. 2012). However, despite that *P.uliginosus* is a hygrophilous species (Štambuk and Erben 2002; Nentwig et al. 2012), in contrast to other species of this genus (e.g., *Piratapiscatorius*, *Piratapiraticus*, *Piratatenuitarsis*, Pirata (Piratula) hygrophilus and Pirata (Piratula) latitans), *P.uliginosus* is least depending on high humidity and can occur in quite dry situations (Roberts 1985, 1996; Harvey et al. 2002b; Almquist 2005; Nentwig et al. 2012).	**Hygrophilous** | Xerophilous; Photophilous | Sciophilous; Habitat generalist *Sub-group*: Bog species
	*Piratulahygrophilus* (Thorell, 1872)	It can be found in damp habitats (Locket and Millidge 1951; Roberts 1996), however it is not normally associated with open water (Harvey et al. 2002b). This species seems to occur mainly in woods (Cristofoli et al. 2010) – it has an affinity to swampy forests and other wet, shady habitats (Kupryjanowicz et al. 1998; Harvey et al. 2002b; Štambuk and Erben 2002; Nentwig et al. 2012). In a couple of studies this species has been reported to be typical for alder forests (Stańska et al. 2000; Štambuk and Erben 2002). It can also be found in *Sphagnum* birch woods very often (Arachnologische Gesellschaft 2018). Nevertheless, *P.hygrophilus* can also occur in wet, open habitats, like damp grasslands (Harvey et al. 2002b; Almquist 2005; Oxbrough et al. 2006). Also, *P.hygrophilus* has been found in fens (Vilbaste 1980), as well as in bogs of Europe (Vilbaste 1980; Šternbergs 1991; Kupryjanowicz et al. 1998; Pommeresche 2002; Rėlys et al. 2002; Spuņģis 2008; Buchholz 2016).	**Hygrophilous** | Xerophilous; Photophilous | **Sciophilous**; Habitat generalist *Sub-group*: Alder forest species
	*Piratulaknorri* (Scopoli, 1763)	It is mainly associated with inland surface waters (Arachnologische Gesellschaft 2018). It inhabits littoral zone of inland surface waterbodies and unvegetated river gravel banks (Nentwig et al. 2012; Arachnologische Gesellschaft 2018), as well as damp areas in woodland (Roberts 1996).	**Hygrophilous** | Xerophilous; Photophilous | Sciophilous; Habitat generalist
Lycosidae	*Piratulalatitans* (Blackwall, 1841)	It is associated with wet habitats which are open to sunshine (Cattin et al. 2003; Oxbrough et al. 2006; Nentwig et al. 2012). This species inhabits open marshes, fens, bogs, reed belts, humid grasslands (Roberts 1996; Harvey et al. 2002b; Buchholz and Schröder 2013). Vilbaste (1980) has found *P.latitans* in Estonian fens, while Kajak et al. (2000) have found it among the dominant spider species in natural fens of Poland. *P.latitans* is less associated with *Sphagnum* bogs than other species of the *Pirata* genus, though it can be found on *Sphagnum* (Harvey et al. 2002b).	**Hygrophilous** | Xerophilous; Photophilous | Sciophilous; Habitat generalist
	*Trochosaruricola* (De Geer, 1778)	It can be found in a range of different wet habitats (Roberts 1996). It occurs in marshes, reed belts, humid grasslands, on the sides of ditches, on shores and elsewhere (Harvey et al. 2002b; Almquist 2005; Buchholz and Schröder 2013). Kajak et al. (2000) found this species in fens, while Rėlys et al. (2002) – in bogs. In Latvia this species has previously been found in Baži bog (Šternbergs 1991; Spuņģis 2008).	**Hygrophilous** | Xerophilous; Photophilous | Sciophilous; Habitat generalist *Sub-group*: Hygrophilous generalist
	*Trochosaspinipalpis* (F. O. Pickard-Cambridge, 1895)	It can be found in a variety of damp habitat types (Roberts 1996), including bogs, fens, wet heathlands and damp meadows (Vilbaste 1980; Kajak et al. 2000; Harvey et al. 2002b; Almquist 2005; Cristofoli et al. 2010; Nentwig et al. 2012). *T.spinipalpis* is usually among the most abundant species in peat bogs of Europe (Kupryjanowicz et al. 1998; Koponen 2002a,b, 2003; Rėlys and Dapkus 2002; Rėlys et al. 2002; Spuņģis 2008).	**Hygrophilous** | Xerophilous; Photophilous | Sciophilous; Habitat generalist *Sub-group*: Hygrophilous generalist
	*Trochosaterricola* Thorell, 1856	It is found in a wide variety of habitats, including woodlands, forest edges, grasslands, heathlands, coastal dunes, sandy shores, vineyards, industrial sites and many other places (Harvey et al. 2002b; Almquist 2005; Isaia et al. 2007; Arachnologische Gesellschaft 2018). *T.terricola* can also be found on mire habitats, especially on bogs (Šternbergs 1991; Kupryjanowicz et al. 1998; Komposch 2000; Pommeresche 2002; Spuņģis 2008). It should be noted, however, that there are some contradictions in the literature about the habitat preferences of this species. Some literature sources say that *T.terricola* shows a preference for drier conditions (Locket and Millidge 1951; Rėlys and Dapkus 2002), while other literature says that it is a hygrophilous species, which can be found in a variety of damp habitats (Roberts 1996; Aakra 2002). In addition, some authors suggest that *T.terricola* is typically a forest spider (Rėlys and Dapkus 2002; Štambuk and Erben 2002). Most authors, however, agree that *T.terricola* is a habitat generalist (e.g., Hänggi et al. 1995; Graham et al. 2003; Mallis and Hurd 2005; Oxbrough et al. 2007).	Hygrophilous | Xerophilous; Photophilous | Sciophilous; **Habitat generalist**
	*Xerolycosanemoralis* (Westring, 1861)	It seems to prefer dry places – it can be found in heathlands, stony chalk grasslands, forest edges and woodland clearings (Locket and Millidge 1951; Roberts 1996; Harvey et al. 2002b; Nentwig et al. 2012). The species also occurs in forests (Cristofoli et al. 2010; Arachnologische Gesellschaft 2018) and bogs (Vilbaste 1980). In addition, *X.nemoralis* favors open, dry and warm areas, which are human-influenced, e.g., sparsely vegetated ground at post-industrial sites (Harvey et al. 2002b), dried peat bogs (Koponen 1979), burned sites (Harvey et al. 2002b; Moretti et al. 2002; Koponen 2005).	Hygrophilous | **Xerophilous**; Photophilous | Sciophilous; Habitat generalist
Miturgidae	*Zoranemoralis* (Blackwall, 1861)	It is associated mainly with forests – it can be found in or near woods (Harvey et al. 2002b), in woodland clearings (Roberts 1996), in moist forest meadows (Almquist 2006). The species can, however, also occur in heather (Locket and Millidge 1951; Almquist 2006) and in other habitats (Arachnologische Gesellschaft 2018).	Hygrophilous | Xerophilous; **Photophilous** | Sciophilous; Habitat generalist *Sub-group*: Ecotonal forest species
Miturgidae	*Zoraspinimana* (Sundevall, 1833)	A widespread and common species (Locket and Millidge 1951; Nentwig et al. 2012). Although it is suggested to be a grassland spider by some authors (Harvey et al. 2002b; Cristofoli et al. 2010), it seems to be indifferent as regards light intensity (Matveinen-Huju et al. 2006), and can be found also in forests (Rėlys and Dapkus 2002; Biteniekyté and Rélys 2008), as well as in a wide variety of other habitats (Locket and Millidge 1951; Roberts 1996; Harvey et al. 2002b; Biteniekyté and Rélys 2008; Arachnologische Gesellschaft 2018). Thus, *Z.spinimana* could be classified as an ubiquitous species (Roberts 1996; Koponen 2002b; Cattin et al. 2003). *Z.spinimana* can also be found in fens (Vilbaste 1980; Kajak et al. 2000) and bogs (Vilbaste 1980; Kupryjanowicz et al. 1998; Koponen 2002b; Rėlys and Dapkus 2002; Rėlys et al. 2002).	Hygrophilous | Xerophilous; Photophilous | Sciophilous; **Habitat generalist**
Oxyopidae	*Oxyopesramosus* (Martini & Goeze, 1778)	It occurs in open, sunny habitats (Arachnologische Gesellschaft 2018), especially in heathlands and similar places, mainly in localities dominated by *Calluna*-heaths (Roberts 1996; Almquist 2005; Aakra and Berggren 2007; Nentwig et al. 2012). The species can also be found in fens and bogs (Vilbaste 1980; Almquist 2005; Aakra and Berggren 2007).	Hygrophilous | Xerophilous; **Photophilous** | Sciophilous; Habitat generalist *Sub-group*: Photophilous generalist
Philodromidae	*Thanatusformicinus* (Clerck, 1757)	It seems to prefer dry habitats (Heimer and Nentwig 1991; Nentwig et al. 2012), especially dry grasslands (Cera 2013; Arachnologische Gesellschaft 2018). It can also be found in forests (Almquist 2006; Arachnologische Gesellschaft 2018), wet heathland (Roberts 1996) and mires (Vilbaste 1980; Harvey et al. 2002b; Koponen 2002a).	Hygrophilous | Xerophilous; **Photophilous** | Sciophilous; Habitat generalist *Sub-group*: Grassland species
	*Tibellusmaritimus* (Menge, 1875)	It occurs in both humid and dry, but sunny habitats (Nentwig et al. 2012). The main habitat types of *T.maritimus* seems to be seashores, coastal sand dunes and marshes with *Carex* and *Cladiummariscus* (Roberts 1996; Bonte et al. 2002; Gajdoš and Toft 2002; Duffey 2005; Almquist 2006). The species can also be found in fens (Vilbaste 1980; Kajak et al. 2000; Cera et al. 2010) and bogs (Vilbaste 1980). *T.maritimus* is usually found close to the sea, however, it can also be detected further inland (Roberts 1996; Duffey 2005).	Hygrophilous | Xerophilous; **Photophilous** | Sciophilous; Habitat generalist *Sub-group*: Coastal species
	*Tibellusoblongus* (Walckenaer, 1802)	It occurs in situations similar to those of *T.maritimus* (Locket and Millidge 1951), however *T.oblongus* is commoner inland and in damper habitats (Roberts 1996). *T.oblongus* can be found in a variety of dry and damp sunny habitats, including seashores, coastal dunes and grasslands of most types (Hänggi et al. 1995; Harvey et al. 2002b; Almquist 2006; Cera et al. 2010; Nentwig et al. 2012; Cera 2013). The species can also occur on fens (Vilbaste 1980; Cera et al. 2010) and bogs (Vilbaste 1980).	Hygrophilous | Xerophilous; **Photophilous** | Sciophilous; Habitat generalist *Sub-group*: Photophilous generalist
Phrurolithidae	*Phrurolithusfestivus* (C. L. Koch, 1835)	It can be found in grasslands, dune heaths, quarries, gardens, and in a variety of similar situations, in both dry and wet conditions (Roberts 1996; Koponen 2000; Harvey et al. 2002b; Almquist 2006; Batáry et al. 2008). Koponen (2005) has recorded it at the burned forest. This species has been found also on bogs (Vilbaste 1980; Rėlys et al. 2002).	Hygrophilous | Xerophilous; **Photophilous** | Sciophilous; Habitat generalist *Sub-group*: Photophilous generalist
Pisauridae	*Dolomedesfimbriatus* (Clerck, 1757)	It occurs in wet, swampy areas (Cattin et al. 2003; Oxbrough et al. 2006; Nentwig et al. 2012). The main habitat of this species is *Sphagnum* bogs and pools, however it can also inhabit moist meadows, alluvial forests, water margins of ditches, ponds, streams and other habitats (Harvey et al. 2002b; Almquist 2005; Nentwig et al. 2012). The literature suggests that *D.fimbriatus* is being found only in those swamps or streams which do not dry up, because this species needs permanent pools of water (Locket and Millidge 1951; Roberts 1996). *D.fimbriatus* can be found in fens and bogs of Europe (Vilbaste 1980; Kajak et al. 2000; Koponen 2002b; Rėlys et al. 2002).	**Hygrophilous** | Xerophilous; Photophilous | Sciophilous; Habitat generalist *Sub-group*: Semi-aquatic species
Pisauridae	*Dolomedesplantarius* (Clerck, 1757)	A species that is very rarely found (Nentwig et al. 2012), and is thought to be in decline throughout Europe (Collins and Wells 1987). This species is associated with damp places such as mires, wet meadows, ponds, banks of rivers, lakes and ditches (Andrušaitis 1998; Holec 2000; Almquist 2005). The main habitats of *D.plantarius*, however, seems to be fens (Collins and Wells 1987; Helsdingen 1993; Roberts 1996; Andrušaitis 1998) and the littoral zone of inland surface waterbodies (Holec 2000; Arachnologische Gesellschaft 2018). *D.plantarius* strongly depends on the presence of water – a permanent, whole year round water surface is obligatory for this species (Helsdingen 1993).	**Hygrophilous** | Xerophilous; Photophilous | Sciophilous; Habitat generalist *Sub-group*: Semi-aquatic species
	*Pisauramirabilis* (Clerck, 1757)	It is common almost everywhere (Locket and Millidge 1951; Cattin et al. 2003), but seems to prefer open habitats (Nentwig et al. 2012). It can be found in grasslands, heathlands, open woods, woodland clearings, gardens and other places (Locket and Millidge 1951; Roberts 1996; Harvey et al. 2002b; Almquist 2005; Arachnologische Gesellschaft 2018). The species has been found also in fens and bogs (Vilbaste 1980).	Hygrophilous | Xerophilous; **Photophilous** | Sciophilous; Habitat generalist *Sub-group*: Photophilous generalist
Salticidae	*Euophrysfrontalis* (Walckenaer, 1802)	It is the commonest species of the genus (Roberts 1996) which can be found in various habitats (Harvey et al. 2002b; Arachnologische Gesellschaft 2018), including forests, meadows and bogs (Vilbaste 1980; Roberts 1996; Rėlys et al. 2002; Almquist 2006). In Latvia this species has previously been found in Baži bog (Šternbergs 1991; Spuņģis 2008).	Hygrophilous | Xerophilous; Photophilous | Sciophilous; **Habitat generalist**
	*Evarchaarcuata* (Clerck, 1757)	It can be found mostly in open, moist habitats (Cattin et al. 2003; Nentwig et al. 2012). It occurs mainly on heathland in damp areas (Roberts 1996; Harvey et al. 2002b), although it can also be found on dry heathland (Harvey et al. 2002b; Nentwig et al. 2012). *E.arcuata* can occur also in meadows (Nyffeler and Benz 1988; Almquist 2006), fens (Vilbaste 1980; Cera et al. 2010) and bogs (Vilbaste 1980; Šternbergs 1991; Rėlys et al. 2002; Spuņģis 2008).	**Hygrophilous** | Xerophilous; **Photophilous** | Sciophilous; Habitat generalist
	*Heliophanuscupreus* (Walckenaer, 1802)	It seems to prefer sunny conditions (Harvey et al. 2002b; Arachnologische Gesellschaft 2018). Overall, however, it can be found in a variety of situations – meadows, woods, forest edges, glades, raised bogs, shingle beaches etc. (Roberts 1996; Harvey et al. 2002b; Almquist 2006; Nentwig et al. 2012). It can also be found in disturbed habitats such as wastelands and quarries (Harvey et al. 2002b).	Hygrophilous | Xerophilous; **Photophilous** | Sciophilous; Habitat generalist
	*Leptorchestesberolinensis* (C. L. Koch, 1846)	It occurs on the bark of trees, on fences, on sunny walls, buildings and on other artificial habitats (Roberts 1996; Nentwig et al. 2012; Arachnologische Gesellschaft 2018).	Hygrophilous | **Xerophilous**; **Photophilous** | Sciophilous; Habitat generalist
	*Marpissaradiata* (Grube, 1859)	It is associated with wet habitats – it can be found in swamps with *Cladiummariscus* and *Carexelata*, on shores of lakes among *Irispseudacorus* (Almquist 2006), on cattail in still water (Nentwig et al. 2012), in fens (Vilbaste 1980; Cera et al. 2010) and bogs (Vilbaste 1980). According to Holec (2000)*M.radiata* is a specialist of the eulittoral zone. Overall, in the continental Europe this species is widespread in wet habitats generally, while in the United Kingdom it seems to be confined to fens (Harvey et al. 2002b).	**Hygrophilous** | Xerophilous; Photophilous | Sciophilous; Habitat generalist *Sub-group*: Hygrophilous generalist
	*Sibianoraurocinctus* (Ohlert, 1865)	It does not appear to be restricted to any particular habitat type apart from the need for dry, warm and sparsely vegetated places (Heimer and Nentwig 1991; Harvey et al. 2002b; Nentwig et al. 2012; Arachnologische Gesellschaft 2018). The species occurs among short vegetation (grass, heather) and amongst stones (Locket and Millidge 1951; Roberts 1996; Harvey et al. 2002b). This species can also be found in human-influenced sites such as sand or chalk quarries and post-industrial sites (Harvey et al. 2002b).	Hygrophilous | **Xerophilous**; **Photophilous** | Sciophilous; Habitat generalist
Salticidae	*Sitticuscaricis* (Westring, 1861)	It seems to have an affinity for swampy areas (Roberts 1996; Harvey et al. 2002b). It has been found in fens, bogs, *Carex*-swamps, damp meadows and moors (Vilbaste 1980; Kajak et al. 2000; Almquist 2006; Nentwig et al. 2012).	**Hygrophilous** | Xerophilous; Photophilous | Sciophilous; Habitat generalist
	*Synagelesvenator* (Lucas, 1836)	It occurs in dry and warm localities, amongst low vegetation, on sandy to rocky ground (Nentwig et al. 2012). This species has mainly been found in sand dunes on the coast and among similar vegetation in fens (Locket and Millidge 1951; Roberts 1996; Harvey et al. 2002b; Duffey 2005). Gajdoš and Toft (2002) recorded this species in dune habitats on the Danish North Sea coast, while Perttula (1984) found it on the coastal sand dunes in Finland. Cera et al. (2010) have also detected this species in a couple of coastal habitats in Latvia. *S.venator* can also be found in birch woods, bogs, fens, on cultivated land, on walls of buildings and on other artificial habitats (Roberts 1996; Almquist 2006; Nentwig et al. 2012; Arachnologische Gesellschaft 2018).	Hygrophilous | **Xerophilous**; Photophilous | Sciophilous; Habitat generalist
	*Talaveraaequipes* (O. Pickard-Cambridge, 1871)	It occurs mainly in dry, warm, sunny habitats with bare surfaces (Harvey et al. 2002b; Nentwig et al. 2012). It has been found in dune heaths, grasslands, sandy or stony banks, quarries and old railway embankments (Harvey et al. 2002b; Duffey 2005; Almquist 2006). It has been found also in fens and bogs (Vilbaste 1980).	Hygrophilous | **Xerophilous**; **Photophilous** | Sciophilous; Habitat generalist
Sparassidae	*Micrommatavirescens* (Clerck, 1757)	It might be found in quite different habitats (Cattin et al. 2003; Arachnologische Gesellschaft 2018). According to Roberts (1996) and Harvey et al. (2002b) it prefers damp sheltered woodlands and woodland clearings, but according to Nentwig et al. (2012) the species prefers sunny and warm conditions. *M.virescens* has been found also on fens (Vilbaste 1980; Cera et al. 2010) and bogs (Vilbaste 1980; Biteniekyté, Rélys 2008).	Hygrophilous | Xerophilous; Photophilous | Sciophilous; **Habitat generalist**
Tetragnathidae	*Pachygnathaclercki* Sundevall, 1823	It seems to occur mostly near water, e.g., at the edges of ponds, rivers and streams (Harvey et al. 2002b; Nentwig et al. 2012). This species has been found, for example, in damp meadows (Almquist 2005; Nentwig et al. 2012), bogs (Vilbaste 1980; Rėlys et al. 2002), fens (Vilbaste 1980; Kajak et al. 2000), and swamp forests (Stańska et al. 2000).	**Hygrophilous** | Xerophilous; Photophilous | Sciophilous; Habitat generalist
	*Tetragnathanigrita* Lendl, 1886	It is most commonly found on trees and shrubs (Roberts 1996; Harvey et al. 2002b; Nentwig et al. 2012). Interestingly, that Glime and Lissner (2013) suggest that this species is largely confined to branches of trees growing on *Sphagnum* bogs and fens, and is only rarely found on the same tree species growing outside bogs and fens. In other literature, however, it is stated that *T.nigrita* can be found on trees in damp woodland (Nentwig et al. 2012), on trees that grow on shores, as well as on fruit trees (Almquist 2005). Although the species is most often found near water (Nentwig et al. 2012), it can also be found in drier situations (Harvey et al. 2002b).	**Hygrophilous** | Xerophilous; Photophilous | Sciophilous; Habitat generalist
Theridiidae	*Crustulinaguttata* (Wider, 1834)	It can be found in both deciduous and pine forests (Roberts 1996; Harvey et al. 2002a; Almquist 2005; Arachnologische Gesellschaft 2018), as well as in open habitats such as meadows (Almquist 2005; Matveinen-Huju et al. 2006). *C.guttata* occurs in drier situations than *C.sticta* – while *C.sticta* prefers wet habitats, *C.guttata* can be usually found on dry, sandy soils (Locket and Millidge 1953; Roberts 1996; Harvey et al. 2002a; Bonte et al. 2003).	Hygrophilous | **Xerophilous**; Photophilous | Sciophilous; Habitat generalist
	*Crustulinasticta* (O. Pickard-Cambridge, 1861)	It lives in wet swampy places such as fens and bogs (Locket and Millidge 1953; Vilbaste 1980; Roberts 1996; Almquist 2005; Nentwig et al. 2012). This species has also been found in several bogs of Latvia (Šternbergs 1991; Spuņģis 2008). Also, *C.sticta* has been recorded from damp heaths, on shingle and amongst marram on dunes (Harvey et al. 2002a).	**Hygrophilous** | Xerophilous; Photophilous | Sciophilous; Habitat generalist
Theridiidae	*Enoplognathaovata* (Clerck, 1757)	It seems to prefer open and sunny habitats (Harvey et al. 2002a; Nentwig et al. 2012), but still it needs the presence of shrubs, bushes, trees or the vicinity of woods (Almquist 2005; Isaia et al. 2007). This species is typical of open habitats containing low broad-leaved vegetation, for example, road verges, domestic gardens and woodland glades (Harvey et al. 2002a). *E.ovata* might also be found in different kinds of forests, dry grasslands, woodland fringes, vineyards near woods and elsewhere (Isaia et al. 2007; Arachnologische Gesellschaft 2018). The species has also been found in fens (Kajak et al. 2000) and bogs (Vilbaste 1980).	Hygrophilous | Xerophilous; **Photophilous** | Sciophilous; Habitat generalist *Sub-group*: Ecotonal forest species
	*Episinusangulatus* (Blackwall, 1836)	It occurs in a wide variety of habitats – in grasslands, mires, woodland clearings, forests etc. (Harvey et al. 2002a; Rėlys and Dapkus 2002; Arachnologische Gesellschaft 2018). Overall, however, it seems to prefer damp situations (Harvey et al. 2002a; Oxbrough et al. 2006; Nentwig et al. 2012). The species is usually found on shrubs and bushes (Heimer and Nentwig 1991; Almquist 2005; Nentwig et al. 2012).	**Hygrophilous** | Xerophilous; Photophilous | Sciophilous; Habitat generalist
	*Euryopisflavomaculata* (C. L. Koch, 1836)	It is reported to be found in damp or boggy places (Locket and Millidge 1953; Roberts 1996; Oxbrough et al. 2006), however, in central Europe it is mainly found in dry habitats, for example, in chalk grasslands, heathlands, coastal dunes (Bonte et al. 2003, 2004; Arachnologische Gesellschaft 2018). The species can also occur in different types of forests – coniferous, deciduous, as well as in mixed forests (Nentwig et al. 2012; Arachnologische Gesellschaft 2018). Koponen (2005) has recoraded *E.flavomaculata* at the burned forest in Finland. In Europe, this species can also be found on fens (Kajak et al. 2000) and bogs (Vilbaste 1980; Kupryjanowicz et al. 1998; Koponen 2002b; Rėlys et al. 2002; Spuņģis 2008).	**Hygrophilous** | Xerophilous; Photophilous | Sciophilous; Habitat generalist *Note*: Preferred habitats differ geographically
	*Neottiurabimaculata* (Linnaeus, 1767)	It seems to be able to live under variable conditions (Harvey et al. 2002a; Nentwig et al. 2012; Arachnologische Gesellschaft 2018). Most records of this species, however, have been from open habitats, especially meadows (Nyffeler and Benz 1988; Matveinen-Huju et al. 2006). Heimer and Nentwig (1991) suggest that this species occurs mainly in roadsides.	Hygrophilous | Xerophilous; **Photophilous** | Sciophilous; Habitat generalist
	*Phyllonetaimpressa* (L. Koch, 1881)	It can be found in forest edges, meadows, heathlands, ruderal areas and in other open places (Almquist 2005; Nentwig et al. 2012; Arachnologische Gesellschaft 2018). This species occurs also in disturbed habitats such as gardens, arable land, intensively grazed grasslands (Almquist 2005; Horváth et al. 2009; Arachnologische Gesellschaft 2018).	Hygrophilous | Xerophilous; **Photophilous** | Sciophilous; Habitat generalist
	*Robertusinsignis* O. Pickard-Cambridge, 1908	It lives in permanent contact with water, and can be found in marshes (Almquist 2005), in very damp meadows (Nentwig et al. 2012) and in fens (Vilbaste 1980; Kajak et al. 2000).	**Hygrophilous** | Xerophilous; Photophilous | Sciophilous; Habitat generalist
	*Theridionvarians* Hahn, 1833	It is found in a variety of different habitats, for example, in forests, grasslands, hedgerows, woodland fringes, mires and in other places (Arachnologische Gesellschaft 2018). This species can be found mainly on trees and shrubs, and also on other structures, for example, buildings and walls (Locket and Millidge 1953; Roberts 1996; Harvey et al. 2002a; Nentwig et al. 2012).	Hygrophilous | Xerophilous; Photophilous | Sciophilous; **Habitat generalist**
Thomisidae	*Ozyptilabrevipes* (Hahn, 1826)	It is usually found in damp, marshy areas (Locket and Millidge 1951; Roberts 1996). It has been found in marshes, in damp alder forests and near the sea (Locket and Millidge 1951; Vilbaste 1980; Harvey et al. 2002b; Almquist 2006). It can, however, also be found in heathlands, grasslands and other drier habitats (Harvey et al. 2002b).	**Hygrophilous** | Xerophilous; Photophilous | Sciophilous; Habitat generalist
Thomisidae	*Ozyptilatrux* (Blackwall, 1846)	It has a wide habitat niche (Roberts 1996; Harvey et al. 2002b) – it is indifferent as regards light intensity and as regards moisture (Matveinen-Huju et al. 2006). Locket and Millidge (1951) propose that this is perhaps the commonest species of the genus. *O.trux* occurs in all types of wet and dry grasslands, coastal dunes and sandy shores, open pine woods, edges of deciduous forests, open agricultural habitats and other places (Harvey et al. 2002b; Almquist 2006; Cristofoli et al. 2010; Arachnologische Gesellschaft 2018). The species has also been found in fens (Vilbaste 1980; Kajak et al. 2000) and bogs (Vilbaste 1980; Spuņģis 2008).	Hygrophilous | Xerophilous; Photophilous | Sciophilous; **Habitat generalist**
	*Xysticusbifasciatus* C. L. Koch, 1837	It is found in habitats with good exposure to the sun (Roberts 1996). The main habitats of the species are dry grasslands and heathland (Harvey et al. 2002b; Almquist 2006; Nentwig et al. 2012; Arachnologische Gesellschaft 2018). It can, however, occur also in fens (Cera et al. 2010) and bogs (Vilbaste 1980).	Hygrophilous | **Xerophilous**; **Photophilous** | Sciophilous; Habitat generalist
	*Xysticuschippewa* Gertsch, 1953	It can be found in moist habitats – fens, bogs, flood plains and damp meadows (Vilbaste 1980; Almquist 2006; Nentwig et al. 2012).	**Hygrophilous** | Xerophilous; Photophilous | Sciophilous; Habitat generalist
	*Xysticuscristatus* (Clerck, 1757)	It is the commonest and most widespread species of the genus (Locket and Millidge 1951; Roberts 1996). Large numbers of this species can be found in grasslands (both damp and dry) and habitats which have undergone some degree of disturbance, for example, quarries and agricultural fields (Harvey et al. 2002b; Almquist 2006). *X.cristatus* is also found on fens (Vilbaste 1980; Kajak et al. 2000) and bogs (Vilbaste 1980; Koponen 2002b; Rėlys et al. 2002). Some literature sources say that *X.cristatus* is a generalist which can be found in almost every habitat type (Aakra 2000; Nentwig et al. 2012), however, other authors suggest that this species is shade-intolerant and thus is rare in shaded habitats (Harvey et al. 2002b; Rėlys and Dapkus 2002b; Oxbrough et al. 2006).	Hygrophilous | Xerophilous; Photophilous | Sciophilous; **Habitat generalist**
	*Xysticuslineatus* (Westring, 1851)	It inhabits damp habitats, for example, damp deciduous woods, bog-forest-like habitats, shores with pebbles (Almquist 2006; Nentwig et al. 2012). It has been found in fens and bogs as well (Vilbaste 1980; Koponen 2002a; Rėlys et al. 2002).	**Hygrophilous** | Xerophilous; Photophilous | Sciophilous; Habitat generalist
	*Xysticusulmi* (Hahn, 1831)	It can be found in damp, marshy habitats (Locket and Millidge 1951; Roberts 1996), and is preferring those wet habitats which are open (Rėlys and Dapkus 2002; Matveinen-Huju et al. 2006; Oxbrough et al. 2006). *X.ulmi* can be found in grasslands, shores, cultivated land, roadside verges etc. (Heimer and Nentwig 1991; Harvey et al. 2002b; Almquist 2006). The species inhabits also mires (Almquist 2006; Arachnologische Gesellschaft 2018), including fens (Vilbaste 1980; Kajak et al. 2000; Cera et al. 2010) and bogs (Vilbaste 1980; Rėlys et al. 2002).	**Hygrophilous** | Xerophilous; **Photophilous** | Sciophilous; Habitat generalist

**Figure 2.**
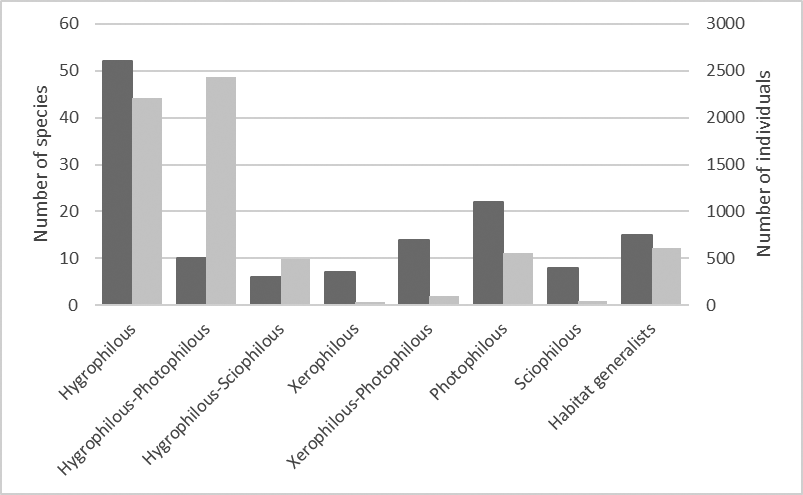
The proportional spider ecological group composition in the calcareous fens of Latvia by the number of species (dark grey columns) and by the number of individuals (light grey columns).

The spider ecological group composition in the studied calcareous fens and the number of spider species and individuals within each group is given in Figure 2. The most species-rich and the most abundant ecological group was hygrophilous species – more than a half of all spider species and individuals collected in the present study could be classified as hygrophilous (if including also hygrophilous-photophilous and hygrophilous-sciophilouspecies). Photophilous species (including photophilous-hygrophilous and photophilous-xerophilous) was another large group in the studied fens – overall, 46 of our collected spider species (34% of all spiders) and 3088 individuals (48%) could be classified as photophilous species. The rest of the ecological groups, xerophilous, sciophilous, and habitat generalists, were represented by a rather low number of species and individuals.

## Discussion

In the present study we investigated the spider fauna of the calcareous fens of the coastal lowland of Latvia. The full species list of the calcareous fen spiders is given in Appendix 1. The main purpose of the present study was to clarify the habitat preferences of the spider species collected during our investigations in the calcareous fens. The habitat preferences of each spider species are described in Table 3.

The arachnofauna of the studied calcareous fens consisted of a wide spectrum of different spider ecological groups. The vast majority of the spider species and individuals found in the fens were hygrophilous or photophilous or hygrophilous-photophilous. The dominance of these groups in the studied habitats is quite logical since all our studied fens were moist, sun-exposed habitats. Nevertheless, the fen arachnofauna consisted also of different other spider ecological groups, including even some groups which do not normally inhabit wet and alkaline environments, i.e., xerophilous and sphagnophilous species. The reason of the presence of such species within calcareous fens might be related with the fact that fens encompass a much broader range of microhabitat variation than other mire types. Fen surface often has a variable microrelief that consists of hummocks, hollows and pools, and since the tops of the hummocks are much drier than their lower part, they may serve as suitable habitat patches for the xerophilous species. Other researchers have also observed that drought-loving spider species can occasionally be found on raised, dry patches of vegetation within otherwise wet and marshy sites (Roberts 1996; Cattin et al. 2003). Similarly, the presence of sphagnophilous spider species within the studied mires might also be explained by the availability of hummocks. Usually these fen hummocks are dominated by acid-loving plant species (especially *Sphagnum* mosses) which are raised above the water level and thus protected from the influence of the alkaline groundwater (Rydin and Jeglum 2006). Consequently, the sphagnophilous spider species, which normally live in acid environments, especially bogs, and are related with *Sphagnum* mosses (e.g., *Gnaphosanigerrima*, *Pardosasphagnicola*, *Piratapiscatorius*), might also be supported in calcareous fens, since the *Sphagnum*-dominated hummocks may serve as discrete habitat patches for them. These findings are supported by several other researchers who have also discovered that spiders can persist in very small microhabitats (Wing 1984; Foelix 2011; Cobbold and MacMahon 2012).

Our study also showed that not only the within-habitat diversity but also the features of the landscape (i.e., the composition of the surrounding habitats) might be very important determinants of the spider species composition of the focal habitat. For example, in the studied fens we have collected several forest species, some of which were detected in fens in a rather great abundance. The occurrence of these forest-related species in our fen habitats could be associated with the fact that all our studied fens were surrounded by forested habitats. Similarly, the presence of coastal and halophilous species within our studied fens might also be largely explained by the proximity of appropriate habitats, since all the studied fens lie in the coastal lowland. Other researchers have also suggested that spider composition of a particular habitat is influenced by the quality of adjacent habitats (e.g., Uetz et al. 1999; Cobbold and MacMahon 2012). In addition, the quantity of nearby habitats also seems to be important: Gallé et al. (2011), for example, discovered that the number of forest specialist species increases in open habitats with increasing proportion of surrounding forests. Thereby, we must emphasise that different spatial scales should always be taken into account in the arachnological investigations, since not only local but also landscape variables could affect the spider fauna of the studied habitat.

Spiders in the present study were sampled by two different techniques, pitfall trapping and sweep netting. Both these methods are among the most popular techniques in spider surveys while pitfall traps have been used extensively for studies on surface-dwelling spiders (e.g., Rėlys et al. 2002; Koponen 2003; Seyfulina 2005; Fetykó 2008), the sweep-net is perhaps the most widely used piece of equipment for sampling spiders from vegetation (e.g., Turnbull 1960; Seyfulina 2005; Fetykó 2008; Horváth et al. 2009). It has been shown that pitfall trapping and sweep netting target different species (Samu and Sarospataki 1995). This was also true in our study: overall, quite different spider species (and even families) were collected with each of these methods (see Appendix 1). We need to emphasize, however, that it is quite hard to compare the obtained data, since using various methods in different sites may influence the results. Other studies have shown that the efficiency of pitfall trapping and sweep netting varies greatly with the structure of the surrounding vegetation (Henderson 2003; Sutherland 2006; Samways et al. 2010). Greenslade (1964), for example, has found that taller vegetation in the vicinity of the pitfall traps hinders invertebrate movement. The sweep netting possess some problems as well – although this method can be used on most vegetation, it is ineffective in some vegetation types, e.g. tall reeds, very short vegetation or flattened vegetation (Sutherland 2006; Henderson 2003). Also, sweep netting is relatively ineffective on sparsely vegetated ground (Sutherland 2006). Thus, we must conclude that it is very difficult to compare catches between different calcareous fens, since our studied fens differed quite greatly from each other in terms of the plant species composition and vegetation height (Štokmane et al. 2013; Štokmane and Spuņģis 2014, 2016). Furthermore, pitfall trapping and sweep netting tend to collect spider species that exhibit different foraging strategies. For example, pitfall traps collect mostly surface-living spiders with an active hunter lifestyle, e.g., many lycosids (Topping and Sunderland 1992; Mallis and Hurd 2005), however, some ground web builders such as those from the family Linyphiidae, can also be adequately sampled (Coyle 1981; Standen 2000). Pitfall traps will not efficiently sample spiders which inhabit the upper vegetation layers (Standen 2000). Sweep netting, on the contrary, is used to catch spiders which occur on the top of the vegetation (Southwood and Henderson 2000). This method is appropriate in low vegetation (Sutherland 2006) and it only collects those spiders that do not fall off on the approach of the collector (Henderson 2003). The sweep net captures primarily aerial web builders (e.g., Araneidae), however aerial hunters could also be collected (Coyle 1981). Overall, it can be concluded that pitfall trapping and sweep netting are methods that successfully complement each other.

In the present study we have also sampled several new spider species for the fauna of Latvia. Recording new species could mainly be explained by insufficient studies in calcareous fens, therefore we suggest that these habitats should be investigated further. In the future studies it would be worthwhile to use a combination of different other spider collection methods (e.g., hand collecting, beating, sieving, suction sampling, etc.) so that a greater variety of microhabitats is accessed. Also, it would be desirable to extend the sampling season throughout the spring, summer and autumn as well as to include both day and night collection, since it is known that spiders exhibit different seasonal and diel activity patterns (Coddington et al. 1996; Marc et al. 1999). Ideally, if the researchers could follow a standardized and optimized sampling protocol when collecting spiders (such as already-existing methodology prepared by Cardoso (2009)), because this could allow future studies in cooperation between different research teams.

The main conclusion from the present study is that calcareous fens are very diverse habitats not only structurally and floristically but also from the arachnofaunistic point of view. Our study showed that calcareous fens contain a very wide range of different spider species, including not only those that need wet and open habitats but also those that prefer other types of microhabitats (e.g., dry, shady, acid, salty, etc.). Besides, we found that along with the terrestrial spider ecological groups, calcareous fens can harbour also semi-aquatic and even aquatic spider species. Overall, however, calcareous fens are poorly investigated habitats, and therefore many spider species might still be undiscovered in this unique mire habitat type. Thereby, in order to get a more accurate picture of the spider fauna of the calcareous fens, these habitats should definitely be investigated further.

## Appendix 1

**Table:** List of spider species and the number of adult individuals caught in the calcareous fens of Latvia by pitfall trapping and/or sweep netting in the summers of three consecutive years (2010, 2011, 2012). Genera and species are sorted alphabetically within each family. Morphospecies are excluded from the table. Abbreviations of the fens: A – Apšuciems, K – Kaņieris, E-1 – Engure-1, E-2 – Engure-2, P – Platene, Ķ – Ķirba, J – Ječi, V – Vītiņi, S – Slītere. Spider species which are new for the fauna of Latvia are marked with asterisk (*).

Family	Species	Fens 2010 (pitfalls)					Fens 2011 (sweep)								Apšuciems 2012	
		A	K	E-1	E-2	P	A	K	E	P	V	S	Ķ	J	sweep	pitfalls
Agelenidae	*Agelenalabyrinthica* (Clerck, 1757)				2	2										2
Araneidae	*Araneusalsine* (Walckenaer, 1802)														1	
	*Araneusdiadematus* Clerck, 1757						3						1		3	
	*Araneusquadratus* Clerck, 1757														3	
	*Araniellacucurbitina* (Clerck, 1757)														3	
	*Argiopebruennichi* (Scopoli, 1772)							1			2		2			
	*Larinioidescornutus* (Clerck, 1757)											1				
	*Mangoraacalypha* (Walckenaer, 1802)														1	
	*Neosconaadianta* (Walckenaer, 1802)														8	
	*Singahamata* (Clerck, 1757)														8	
Clubionidae	*Clubionagermanica* Thorell, 1871														6	
	*Clubionareclusa* O. Pickard-Cambridge, 1863					2										
	*Clubionastagnatilis* Kulczyński, 1897					2										
	*Clubionasubsultans* Thorell, 1875															2
Dictynidae	*Argennasubnigra* (O. Pickard-Cambridge, 1861)			3												
	*Argyronetaaquatica* (Clerck, 1757)				2	5										2
Eutichuridae	*Cheiracanthiumerraticum* (Walckenaer, 1802)										1					1
	*Cheiracanthiumpunctorium* (Villers, 1789) *														2	1
Gnaphosidae	*Drassodeslapidosus* (Walckenaer, 1802)		5	1	10											
	*Drassodespubescens* (Thorell, 1856)	2	4	1												
	*Drassylluslutetianus* (L. Koch, 1866)	12	8	24	24	69										3
	*Drassylluspraeficus* (L. Koch, 1866)	1														
Gnaphosidae	*Drassylluspusillus* (C. L. Koch, 1833)		10		1											
	*Gnaphosabicolor* (Hahn, 1833)		1		20											
	*Gnaphosalapponum* (L. Koch, 1866) *	1														
	*Gnaphosanigerrima* L. Koch, 1877 *	1			21											
	*Haplodrassusmoderatus* (Kulczyński, 1897)															1
	*Haplodrassussignifer* (C. L. Koch, 1839)		3	2												2
	*Haplodrassussilvestris* (Blackwall, 1833)			9	7	14										1
	*Micariapulicaria* (Sundevall, 1831)			2		8										
	*Poecilochroavariana* (C. L. Koch, 1839)				1											
	*Zelotesclivicola* (L. Koch, 1870)		1													
	*Zeloteslatreillei* (Simon, 1878)	5	4	4	8											1
	*Zelotessubterraneus* (C. L. Koch, 1833)															1
Hahniidae	*Antisteaelegans* (Blackwall, 1841)	139	1	35	124	28										104
Linyphiidae	*Agynetamollis* (O. Pickard-Cambridge, 1871)															2
	*Agynetasubtilis* (O. Pickard-Cambridge, 1863)															1
	*Allomengeavidua* (L. Koch, 1879)															16
	*Bathyphantesgracilis* (Blackwall, 1841)			4												12
	*Bathyphantesnigrinus* (Westring, 1851)															1
	*Bathyphantesparvulus* (Westring, 1851) *	44	32	16	8	13										11
	*Bolyphanthesalticeps* (Sundevall, 1833)		1													
	*Centromerussemiater* (L. Koch, 1879) *															2
	*Ceratinellabrevipes* (Westring, 1851)	3														
	*Diplostylaconcolor* (Wider, 1834)	2		1	3											
	*Dismodicuselevatus* (C. L. Koch, 1838)					3										
	*Erigonearctica* (White, 1852)															4
	*Erigoneatra* Blackwall, 1833					36										
	*Erigonedentipalpis* (Wider, 1834)						2									
	*Erigonellahiemalis* (Blackwall, 1841)	20		1		46										2
	*Erigonellaignobilis* (O. Pickard-Cambridge, 1871)				10	13										
	*Floroniabucculenta* (Clerck, 1757)															2
	*Gnathonariumdentatum* (Wider, 1834)					4				1						
	*Gongylidiellumlatebricola* (O. Pickard-Cambridge, 1871)															3
	*Kaestneriapullata* (O. Pickard-Cambridge, 1863)			1										1		1
	*Linyphiahortensis* Sundevall, 1830														1	
	*Micrargusherbigradus* (Blackwall, 1854)		1		1											
	*Microlinyphiaimpigra* (O. Pickard-Cambridge, 1871) *					1										
	*Microlinyphiapusilla* (Sundevall, 1830)				1			1				1				
	*Nerienemontana* (Clerck, 1757)		3	2	1											
	*Notioscopussarcinatus* (O. Pickard-Cambridge, 1873)				16	4										
	*Pocadicnemispumila* (Blackwall, 1841)		4		2											2
	*Styloctetorcompar* (Westring, 1861)	5		5	2	2										
Liocranidae	*Tallusiaexperta* (O. Pickard-Cambridge, 1871)					1										
	*Tenuiphantescristatus* (Menge, 1866)	1	1													
	*Trichopternoidesthorelli* (Westring, 1861)	4		1		12										
	*Typhochrestusdigitatus* (O. Pickard-Cambridge, 1873)					16										
	*Walckenaeriaalticeps* (Denis, 1952)	3		3	12	12										9
	*Walckenaeriaatrotibialis* (O. Pickard-Cambridge, 1878)	8	1			4										1
	*Walckenaeriavigilax* (Blackwall, 1853)			1	3	15										
	*Agroecadentigera* Kulczyński, 1913				1	1										2
	*Agroecaproxima* (O. Pickard-Cambridge, 1871)															1
	*Liocranoecastriata* (Kulczyński, 1882)	22	2	17	3	9										3
	*Scotinapalliardi* (L. Koch, 1881)		2													
Lycosidae	*Alopecosapulverulenta* (Clerck, 1757)		28		1	1										
	*Arctosaleopardus* (Sundevall, 1833)				17	20										
	*Auloniaalbimana* (Walckenaer, 1805)		2													1
	*Hygrolycosarubrofasciata* (Ohlert, 1865)	5	11	43	3	2										98
	*Pardosafulvipes* (Collett, 1876)	6		19	12	16									4	34
	*Pardosalugubris* (Walckenaer, 1802)		2		2											11
	*Pardosaprativaga* (L. Koch, 1870)	10	24	122	28	571										
	*Pardosaproxima* (C. L. Koch, 1847)					3										
	*Pardosapullata* (Clerck, 1757)	27		26	101	69										3
	*Pardosasaltans* Töpfer-Hofmann, 2000	1														
	*Pardosasphagnicola* (Dahl, 1908)						2			3	2	1	2	3		66
	*Piratapiraticus* (Clerck, 1757)		2													
	*Piratapiscatorius* (Clerck, 1757)	1														
	*Piratatenuitarsis* Simon, 1876 *	16		78	37	204										86
	*Piratauliginosus* (Thorell, 1856)	38	144	29	115	32										28
	*Piratulahygrophilus* (Thorell, 1872)	9		71	51	51										102
	*Piratulaknorri* (Scopoli, 1763)	13		32	3	196										68
	*Piratulalatitans* (Blackwall, 1841)			807												
	*Trochosaruricola* (De Geer, 1778)	1				1										2
	*Trochosaspinipalpis* (F. O. Pickard-Cambridge, 1895)	9	19	13	19	9										
	*Trochosaterricola* Thorell, 1856				1											122
	*Xerolycosanemoralis* (Westring, 1861)															1
Miturgidae	*Zoranemoralis* (Blackwall, 1861)				4											
	*Zoraspinimana* (Sundevall, 1833)	9	18	17	10	7										99
Oxyopidae	*Oxyopesramosus* (Martini & Goeze, 1778)														54	1
Philodromidae	*Thanatusformicinus* (Clerck, 1757)		1													
	*Tibellusmaritimus* (Menge, 1875)			1	1	1	9	12	12	9		3	11	24	6	
	*Tibellusoblongus* (Walckenaer, 1802)														5	
Phrurolithidae	*Phrurolithusfestivus* (C. L. Koch, 1835)	1	12	5	3	1										16
Pisauridae	*Dolomedesfimbriatus* (Clerck, 1757)	14	3	7	6	10	8	27	7	9		1	14	8	409	52
	*Dolomedesplantarius* (Clerck, 1757)							5								
	*Pisauramirabilis* (Clerck, 1757)				1	1									18	3
Salticidae	*Euophrysfrontalis* (Walckenaer, 1802)		1	1												
	*Evarchaarcuata* (Clerck, 1757)	1	2		1		11	7		1		1	2	4	90	13
	*Heliophanuscupreus* (Walckenaer, 1802)														24	
Salticidae	*Leptorchestesberolinensis* (C. L. Koch, 1846) *															9
	*Marpissaradiata* (Grube, 1859)						3	6				4			4	
	*Sibianoraurocinctus* (Ohlert, 1865)															2
	*Sitticuscaricis* (Westring, 1861)			1												
	*Synagelesvenator* (Lucas, 1836)														14	
	*Talaveraaequipes* (O. Pickard-Cambridge, 1871)	1	1	1	3											2
Sparassidae	*Micrommatavirescens* (Clerck, 1757)				1											
Tetragnathidae	*Pachygnathaclercki* Sundevall, 1823					11										1
	*Tetragnathanigrita* Lendl, 1886														6	
Theridiidae	*Crustulinaguttata* (Wider, 1834)	1														
	*Crustulinasticta* (O. Pickard-Cambridge, 1861)	1														
	*Enoplognathaovata* (Clerck, 1757)												1		2	
	*Episinusangulatus* (Blackwall, 1836)			1	1										1	2
	*Euryopisflavomaculata* (C. L. Koch, 1836)	3	6	1	6											10
	*Neottiurabimaculata* (Linnaeus, 1767)	1	1	1	1											
	*Phyllonetaimpressa* (L. Koch, 1881)										2				1	
	*Robertusinsignis* O. Pickard-Cambridge, 1908	1		3	1											
	*Theridionvarians* Hahn, 1833														2	
Thomisidae	*Ozyptilabrevipes* (Hahn, 1826)			1												
	*Ozyptilatrux* (Blackwall, 1846)	10	2	20	24	5										3
	*Xysticusbifasciatus* C. L. Koch, 1837					1										
	*Xysticuschippewa* Gertsch, 1953					1										
	*Xysticuscristatus* (Clerck, 1757)					3										
	*Xysticuslineatus* (Westring, 1851)		1													
	*Xysticusulmi* (Hahn, 1831)		1		1	3	3	3	1	1			1	1	11	2
**Total number of individuals**		**452**	**365**	**1433**	**737**	**1541**	**41**	**62**	**20**	**24**	**7**	**12**	**34**	**41**	**687**	**1033**
**Total number of species**		**40**	**38**	**43**	**52**	**49**	**8**	**8**	**3**	**6**	**4**	**7**	**8**	**6**	**26**	**57**

## Appendix 2

**Table:** The list of all our collected spider species and their occurrence within mire habitats of Europe in other studies. Only those publications in which the full spider species list was available are included. The information about each species is given as presence/absence data. Genera and species are sorted alphabetically within each family.

Family	Species	FENS OF EUROPE			BOGS OF EUROPE						
		Latvia	Estonia	Poland	Latvia	Estonia	Poland	Lithuania		Finland	
		Cera et al. 2010	Vilbaste 1980	Kajak et al. 2000	Šternbergs 1991	Vilbaste 1980	Kupryjanowicz et al. 1998	Rėlys et al. 2002	Rėlys and Dapkus 2002b	Koponen 2002a	Koponen 2002b
Agelenidae	*Agelenalabyrinthica* (Clerck, 1757)					×					
Araneidae	*Araneusalsine* (Walckenaer, 1802)					×					
	*Araneusdiadematus* Clerck, 1757	×				×					
	*Araneusquadratus* Clerck, 1757	×	×	×		×					
	*Araniellacucurbitina* (Clerck, 1757)		×			×					
	*Argiopebruennichi* (Scopoli, 1772)										
	*Larinioidescornutus* (Clerck, 1757)	×		×							
	*Mangoraacalypha* (Walckenaer, 1802)			×					×		
	*Neosconaadianta* (Walckenaer, 1802)			×							
	*Singahamata* (Clerck, 1757)		×	×		×					
Clubionidae	*Clubionagermanica* Thorell, 1871		×			×					
	*Clubionareclusa* O. Pickard-Cambridge, 1863					×					
	*Clubionastagnatilis* Kulczyński, 1897		×	×		×			×		
	*Clubionasubsultans* Thorell, 1875		×			×					
Dictynidae	*Argennasubnigra* (O. Pickard-Cambridge, 1861)			×							
	*Argyronetaaquatica* (Clerck, 1757)		×			×					
Eutichuridae	*Cheiracanthiumerraticum* (Walckenaer, 1802)		×			×					
	*Cheiracanthiumpunctorium* (Villers, 1789)										
Gnaphosidae	*Drassodeslapidosus* (Walckenaer, 1802)			×							
	*Drassodespubescens* (Thorell, 1856)		×					×	×	×	×
	*Drassylluslutetianus* (L. Koch, 1866)		×				×	×	×		
	*Drassylluspraeficus* (L. Koch, 1866)										
	*Drassylluspusillus* (C. L. Koch, 1833)							×	×	×	
	*Gnaphosabicolor* (Hahn, 1833)										
	*Gnaphosalapponum* (L. Koch, 1866)									×	×
	*Gnaphosanigerrima* L. Koch, 1877						×	×	×		×
	*Haplodrassusmoderatus* (Kulczyński, 1897)		×			×		×		×	
	*Haplodrassussignifer* (C. L. Koch, 1839)		×			×	×	×	×	×	×
	*Haplodrassussilvestris* (Blackwall, 1833)							×			
	*Micariapulicaria* (Sundevall, 1831)		×			×		×			
Gnaphosidae	*Poecilochroavariana* (C. L. Koch, 1839)										
	*Zelotesclivicola* (L. Koch, 1870)						×	×	×		
	*Zeloteslatreillei* (Simon, 1878)			×				×	×	×	
	*Zelotessubterraneus* (C. L. Koch, 1833)							×	×		
Hahniidae	*Antisteaelegans* (Blackwall, 1841)		×	×		×	×	×	×	×	×
Linyphiidae	*Agynetamollis* (O. Pickard-Cambridge, 1871)		×			×					
	*Agynetasubtilis* (O. Pickard-Cambridge, 1863)								×		
	*Allomengeavidua* (L. Koch, 1879)			×							
	*Bathyphantesgracilis* (Blackwall, 1841)		×	×				×		×	×
	*Bathyphantesnigrinus* (Westring, 1851)	×									
	*Bathyphantesparvulus* (Westring, 1851)			×						×	
	*Bolyphanthesalticeps* (Sundevall, 1833)		×								
	*Centromerussemiater* (L. Koch, 1879)			×							
	*Ceratinellabrevipes* (Westring, 1851)		×	×		×					
	*Diplostylaconcolor* (Wider, 1834)										
	*Dismodicuselevatus* (C. L. Koch, 1838)	×	×			×		×			
	*Erigonearctica* (White, 1852)										
	*Erigoneatra* Blackwall, 1833	×	×	×		×					×
	*Erigonedentipalpis* (Wider, 1834)		×	×		×					
	*Erigonellahiemalis* (Blackwall, 1841)					×					
	*Erigonellaignobilis* (O. Pickard-Cambridge, 1871)		×								
	*Floroniabucculenta* (Clerck, 1757)								×		
	*Gnathonariumdentatum* (Wider, 1834)		×			×					
	*Gongylidiellumlatebricola* (O. Pickard-Cambridge, 1871)										
	*Kaestneriapullata* (O. Pickard-Cambridge, 1863)	×	×	×		×					
	*Linyphiahortensis* Sundevall, 1830										
	*Micrargusherbigradus* (Blackwall, 1854)				×	×			×		
	*Microlinyphiaimpigra* (O. Pickard-Cambridge, 1871)										
	*Microlinyphiapusilla* (Sundevall, 1830)		×	×		×					
	*Nerienemontana* (Clerck, 1757)					×					
	*Notioscopussarcinatus* (O. Pickard-Cambridge, 1873)		×	×	×	×	×		×		
	*Pocadicnemispumila* (Blackwall, 1841)		×	×		×	×	×	×		
	*Styloctetorcompar* (Westring, 1861)										
	*Tallusiaexperta* (O. Pickard-Cambridge, 1871)			×				×	×	×	
	*Tenuiphantescristatus* (Menge, 1866)							×			
	*Trichopternoidesthorelli* (Westring, 1861)		×			×					
Linyphiidae	*Typhochrestusdigitatus* (O. Pickard-Cambridge, 1873)										
	*Walckenaeriaalticeps* (Denis, 1952)						×	×	×		
	*Walckenaeriaatrotibialis* (O. Pickard-Cambridge, 1878)						×	×	×		×
	*Walckenaeriavigilax* (Blackwall, 1853)			×							
Liocranidae	*Agroecadentigera* Kulczyński, 1913			×				×			×
	*Agroecaproxima* (O. Pickard-Cambridge, 1871)						×	×	×	×	×
	*Liocranoecastriata* (Kulczyński, 1882)			×							
	*Scotinapalliardi* (L. Koch, 1881)				×	×	×	×	×	×	×
Lycosidae	*Alopecosapulverulenta* (Clerck, 1757)			×	×	×	×	×	×	×	×
	*Arctosaleopardus* (Sundevall, 1833)			×		×					
	*Auloniaalbimana* (Walckenaer, 1805)							×	×		
	*Hygrolycosarubrofasciata* (Ohlert, 1865)		×	×		×	×	×			
	*Pardosafulvipes* (Collett, 1876)		×			×					
	*Pardosalugubris* (Walckenaer, 1802)			×		×	×	×			
	*Pardosaprativaga* (L. Koch, 1870)		×	×		×	×	×	×		
	*Pardosapullata* (Clerck, 1757)		×	×		×	×	×	×	×	×
	*Pardosaproxima* (C. L. Koch, 1847)										
	*Pardosasaltans* Töpfer-Hofmann, 2000										
	*Pardosasphagnicola* (Dahl, 1908)		×	×		×	×	×	×	×	×
	*Piratapiraticus* (Clerck, 1757)		×	×		×		×		×	
	*Piratapiscatorius* (Clerck, 1757)		×	×						×	×
	*Piratatenuitarsis* Simon, 1876			×							
	*Piratauliginosus* (Thorell, 1856)			×		×	×	×	×	×	×
	*Piratulahygrophilus* (Thorell, 1872)		×		×	×	×	×	×		
	*Piratulaknorri* (Scopoli, 1763)										
	*Piratulalatitans* (Blackwall, 1841)		×	×							
	*Trochosaruricola* (De Geer, 1778)			×	×			×			
	*Trochosaspinipalpis* (F. O. Pickard-Cambridge, 1895)		×	×		×	×	×	×	×	×
	*Trochosaterricola* Thorell, 1856				×		×				
	*Xerolycosanemoralis* (Westring, 1861)					×					
Miturgidae	*Zoranemoralis* (Blackwall, 1861)										
	*Zoraspinimana* (Sundevall, 1833)		×	×		×	×	×	×		×
Oxyopidae	*Oxyopesramosus* (Martini & Goeze, 1778)		×			×					
Philodromidae	*Thanatusformicinus* (Clerck, 1757)		×			×				×	
	*Tibellusmaritimus* (Menge, 1875)	×	×	×		×					
	*Tibellusoblongus* (Walckenaer, 1802)	×	×			×					
Phrurolithidae	*Phrurolithusfestivus* (C. L. Koch, 1835)					×		×			
Pisauridae	*Dolomedesfimbriatus* (Clerck, 1757)		×	×		×		×	×		×
	*Dolomedesplantarius* (Clerck, 1757)										
	*Pisauramirabilis* (Clerck, 1757)		×			×					
Salticidae	*Euophrysfrontalis* (Walckenaer, 1802)				×	×		×			
	*Evarchaarcuata* (Clerck, 1757)	×	×		×	×		×			
	*Heliophanuscupreus* (Walckenaer, 1802)										
	*Leptorchestesberolinensis* (C. L. Koch, 1846)										
	*Marpissaradiata* (Grube, 1859)	×	×			×					
	*Sibianoraurocinctus* (Ohlert, 1865)										
	*Sitticuscaricis* (Westring, 1861)		×	×		×					
	*Synagelesvenator* (Lucas, 1836)	×	×			×					
	*Talaveraaequipes* (O. Pickard-Cambridge, 1871)		×			×					
Sparassidae	*Micrommatavirescens* (Clerck, 1757)	×	×			×					
Tetragnathidae	*Pachygnathaclercki* Sundevall, 1823		×	×		×		×			
	*Tetragnathanigrita* Lendl, 1886										
Theridiidae	*Crustulinaguttata* (Wider, 1834)		×	×		×		×			
	*Crustulinasticta* (O. Pickard-Cambridge, 1861)		×		×	×					
	*Enoplognathaovata* (Clerck, 1757)			×		×					
	*Episinusangulatus* (Blackwall, 1836)								×		
	*Euryopisflavomaculata* (C. L. Koch, 1836)			×		×	×	×			×
	*Neottiurabimaculata* (Linnaeus, 1767)										
	*Phyllonetaimpressa* (L. Koch, 1881)										
	*Robertusinsignis* O. Pickard-Cambridge, 1908		×	×							
	*Theridionvarians* Hahn, 1833					×					
Thomisidae	*Ozyptilabrevipes* (Hahn, 1826)		×			×					
	*Ozyptilatrux* (Blackwall, 1846)		×	×		×					
	*Xysticusbifasciatus* C. L. Koch, 1837	×				×					
	*Xysticuschippewa* Gertsch, 1953		×								
	*Xysticuscristatus* (Clerck, 1757)		×	×		×		×			×
	*Xysticuslineatus* (Westring, 1851)		×			×		×		×	
	*Xysticusulmi* (Hahn, 1831)	×	×	×		×		×	×		
